# CVD of MoS_2_ single layer flakes using Na_2_MoO_4_ – impact of oxygen and temperature–time-profile†

**DOI:** 10.1039/d3nr03907b

**Published:** 2023-11-02

**Authors:** Romana Alice Kalt, Andrea Arcifa, Christian Wäckerlin, Andreas Stemmer

**Affiliations:** a Nanotechnology Group, ETH Zürich Säumerstrasse 4 CH-8803 Rüschlikon Switzerland kaltro@ethz.ch astemmer@ethz.ch; b Surface Science & Coating Technologies, Swiss Federal Laboratories for Materials Science and Technology (EMPA) Überlandstrasse 129 CH-8600 Dübendorf Switzerland; c Institute of Physics, Swiss Federal Institute of Technology Lausanne (EPFL) Station 3 CH-1015 Lausanne Switzerland; d Laboratory for X-ray Nanoscience and Technologies, Paul-Scherrer-Institute (PSI) CH-5232 Villigen PSI Switzerland

## Abstract

Two-dimensional (2D) materials are of great interest in many fields due to their astonishing properties at an atomic level thickness. Many fundamentally different methods to synthesize 2D materials, such as exfoliation or chemical vapor deposition (CVD), have been reported. Despite great efforts and progress to investigate and improve each synthesis method, mainly to increase the yield and quality of the synthesized 2D materials, most approaches still involve some compromise. Herein, we systematically investigate a chemical vapor deposition (CVD) process to synthesize molybdenum disulfide (MoS_2_) single layer flakes using sodium molybdate (Na_2_MoO_4_), deposited on a silica (SiO_2_/Si) substrate by spin-coating its aqueous solution, as the molybdenum source and sulfur powder as sulfur source, respectively. The focus lies on the impact of oxygen (O_2_) in the gas flow and temperature–time-profile on reaction process and product quality. Atomic force microscopy (AFM), Raman and photoluminescence (PL) spectroscopy, X-ray photoelectron spectroscopy (XPS), and time-of-flight secondary ion mass spectrometry (ToF-SIMS) were used to investigate MoS_2_ flakes synthesized under different exposure times of O_2_ and with various temperature–time-profiles. This detailed study shows that the MoS_2_ flakes are formed within the first few minutes of synthesis and elaborates on the necessity of O_2_ in the gas flow, as well as drawbacks of its presence. In addition, the applied temperature–time-profile highly affects the ability to detach MoS_2_ flakes from the growth substrate when immersed in water, but it has no impact on the flake.

## Abstract 2

In-depth investigation of MoS_2_ flakes synthesized by CVD to derive insights on growth, process parameters, and detachability enabled by water.

## Graphical abstract

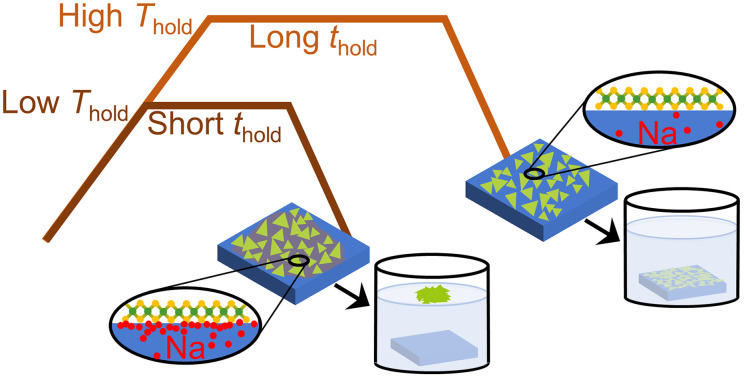

## Introduction

Crystals with a layered structure, like graphite, can generally be mechanically cleaved into single- or very few-layered flakes, analogous to graphene.^1,2^ About two-thirds of the transition metal dichalcogenide (TMDC) crystals consist of such a layered structure and allow fabrication of single layered flakes, commonly called two-dimensional (2D) materials.^1^ These single layered TMDCs possess outstanding chemical and physical properties,^3,4^ making them interesting 2D materials useful as building blocks for a wide range of devices from electronics^5,6^ to catalysis,^7^ and biomedical applications.^8^ Molybdenum disulfide (molybdenite, MoS_2_) is a prominent representative of the layered TMDCs and is widely studied due to its substantial direct band gap,^9,10^ high on/off ratio,^11^ flexibility,^12^ strong photoluminescence,^13,14^ and many more properties.^15^ Next to the fabrication of MoS_2_ single layer flakes by mechanical cleavage, other top-down synthesis methods such as electrochemical exfoliation^16^ and liquid exfoliation in a suitable solvent^17^ or with a pre-intercalation step^18^ were developed to increase the production yield of flakes. However, the latter methods lack control over the lateral size and number of layers, and in addition, transitions in the polytype from the trigonal prismatic (2H) to the octahedral phase (1T) cannot be prevented.^18^ This inhomogeneity in the fabricated flakes gives rise to undesired varying properties of the final product. To tackle these issues, bottom-up approaches such as atomic layer deposition,^19^ molecular beam epitaxy,^20^ and chemical vapor deposition (CVD)^21,22^ have been developed. Among these synthesis methods, CVD is regarded as a very promising technique due to good scalability, low cost, relative simplicity, and better control of the size and thickness of the synthesized MoS_2_ flakes. Nevertheless, control of the synthesis by CVD remains a challenge, and the underlying growth mechanism is not yet fully understood due to a large number of different parameters such as precursors, temperature–time-profile, composition and rate of gas flow, growth substrate material, and pressure, as well as their interdependence.^23,24^ Commonly, synthesis of MoS_2_ flakes by CVD is performed using molybdenum trioxide (MoO_3_) powder as molybdenum (Mo) source, placed in a crucible and partially covered by the growth substrate, and sulfur (S) powder positioned separately in the upstream heating zone. This set-up leads to spatial nonuniformity of the synthesized MoS_2_ flakes on the growth substrate and, therefore, reduced control of the growth process due to the significant local differences in vapor pressure, non-homogeneous diffusion of the precursors, and reaction of vaporized sulfur with MoO_3_ powder, poisoning the Mo-source.^25^ Enhanced process control was achieved using gaseous precursors such as preheated molybdenum hexacarbonyl (Mo(CO)_6_) as Mo-source and diethyl sulfide (S(C_2_H_5_)_2_) or hydrogen sulfide (H_2_S) as S-source.^26–28^ However, H_2_S and Mo(CO)_6_ gases are highly toxic, which limits their wider application, while the organic ligands in S(C_2_H_5_)_2_ get incorporated in the synthesized MoS_2_, reducing the final flake quality.^29,30^ To circumvent the toxicity and inhomogeneous diffusion of the Mo-source in the gas phase when using Mo(CO)_6_ gas or MoO_3_ powder piled up below the growth substrate, the Mo-source can instead be uniformly distributed over the entire growth substrate prior to the synthesis by spin-coating an aqueous Mo-solution.^31,32^ This synthesis method provides an effective approach to synthesize single layered MoS_2_, yet, despite its simplicity, it is seldomly used and lacks detailed description and in-depth investigation of process parameters and their influence on the reaction process and flake quality.

**Fig. 1:**
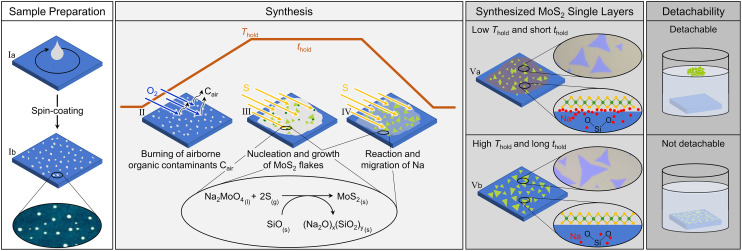
Schematic drawing of the proposed reaction process to form MoS_2_ flakes with different degree of detachability. The subsets, identified by roman numerals, represent the sample preparation through spin-coating (Ia and Ib), the burning of airborne organic contaminants by oxygen (II), Na_2_MoO_4_ conversion to MoS_2_ (III), further reaction of Na with the silica substrate and its migration into the SiO_2_ matrix (IV), and the distinction between water-detachable and non-water-detachable MoS_2_ flakes (Va, Vb). Depending on the *T*–*t*-profile, the flakes sit on a Na-containing and a water-soluble by-product or are in direct contact with the substrate, reducing the detachability. The oval insets show the corresponding AFM measurements, light microscopy images, or schematic illustrations, respectively.

In this work, we investigate the CVD process based on sodium molybdate (Na_2_MoO_4_), spin-coated as aqueous solution onto a silica (SiO_2_/Si) growth substrate. The focus lies on the impact of oxygen (O_2_) in the gas flow and the temperature–time-profile, defined by holding temperature and holding time, on reaction process and product quality. Our synthesis protocol, with O_2_ present in the gas flow in the early stage of the synthesis process, yields homogeneous distributions of high-quality MoS_2_ single layer flakes over a wide range of temperature–time-profiles. Interestingly, for low enough holding temperature and short holding time, MoS_2_ flakes detach from the growth substrate when immersed in water. We propose a reaction process (see Fig. 1), which we validated using atomic force microscopy (AFM), Raman and photoluminescence (PL) spectroscopy as well as X-ray photoemission spectroscopy (XPS) and time-of-flight secondary ion mass spectrometry (ToF-SIMS) to determine flake quality, sample topography, and chemical composition.

## Synthesis and characterization

### Synthesis model

Our synthesis of MoS_2_ single layer flakes by CVD with sodium molybdate (Na_2_MoO_4_) is based on the vapour–liquid–solid (VLS) growth mode.^32,33^ Therein, vaporized sulfur (S) reacts with liquified Na_2_MoO_4_ on the growth substrate to form solid MoS_2_ when the temperature is raised above the specific melting point of S and Na_2_MoO_4_, respectively. Here, we first summarize the synthesis model (Fig. 1 and its subsets identified by roman numerals) and subsequently address key aspects as the requirement of oxygen (O_2_) in the formation of MoS_2_ single layer flakes, process duration, influence of the temperature–time-profile (*T*–*t*-profile), and the role of sodium.

Spin-coating and drying of the aqueous solution of Na_2_MoO_4_ on silica (SiO_2_/Si) lead to formation of small clumps uniformly distributed over the entire substrate, which entails homogeneous distribution of the Mo-source (Ia, Ib). While heating up the tube furnace to a specific temperature (holding temperature, *T*_hold_), the O_2_ in the gas flow burns away any kind of airborne organic contaminants on the growth substrate and enables the growth of triangular flakes (II). When the center of the tube furnace reaches a temperature of 650 °C the sulfur powder, placed in a crucible in the upstream heating zone, starts to melt and evaporates, subsequently being transported to the growth substrate by the gas flow, where it reacts with the liquified Na_2_MoO_4_ at around 690 °C to form MoS_2_ within a very short time frame (III).^32^ With increasing time, only chemical changes related to sodium (Na) originating from the Mo-source take place (IV). At low *T*_hold_ and short holding time (*t*_hold_), which refers to the time frame in which the center of the tube furnace is at *T*_hold_, a thin layer of Na-containing and water-soluble by-product remains between the growth substrate and MoS_2_ flakes (Va). Upon immersion of the growth substrate into water, this layer dissolves, leading to complete detachment of the MoS_2_ flakes. These detached MoS_2_ flakes float on the water surface as a greenish carpet and can be collected by immersing a fresh substrate and picking them up. Samples synthesized at high *T*_hold_ and long *t*_hold_, however, do not exhibit this water-soluble layer due to enhanced migration of the Na-by-product into the substrate (Vb). Consequently, the MoS_2_ flakes are in direct contact with the growth substrate rendering them impossible to be detached by water. This Na-by-product might be composed of sodium oxide (Na_2_O), a possible side-product of the MoS_2_ flake formation, as described elsewhere.^34^ Na_2_O exhibits both temperature-dependent reactivity with SiO_2_, forming sodium silicate glasses, and migration into SiO_2_, where it becomes diluted within the silica glass matrix, consonant with our findings.^35^

### Requirement of oxygen

**Fig. 2:**
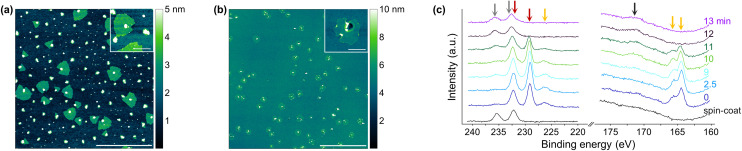
(a) AFM images of a sample synthesized at 710 °C for 6 min without O_2_ in the gas flow during synthesis, hampering the growth of triangular MoS_2_ single layer flakes. The scale bar represents 2 μm and 400 nm (inset), respectively. (b) AFM images of a sample synthesized at 710 °C for 6 min with O_2_ exposure for 13 min during synthesis. The scale represents 5 μm and 400 nm (inset), respectively. (c) High-resolution XP-spectra of Mo 3d, S 2s, and S 2p core level peak regions of samples synthesized at 710 °C for 6 min and various exposure times of O_2_. Sulfates (black arrow) and Mo^6+^ (grey arrows) were detected in samples exposed to oxygen for prolonged time during synthesis, while the Mo^4+^-signals (red arrows) and the MoS_2_-sulfur signals (orange arrows) disappear. This observation points to the oxidation of the *in situ* formed MoS_2_ flakes when the samples were exposed to O_2_ at elevated temperatures for too long.

The composition of the gas flow present during CVD plays a crucial role for successful synthesis of triangular MoS_2_ single layer flakes with Na_2_MoO_4_ uniformly distributed on a SiO_2_/Si substrate by spin-coating its aqueous solution. Kim *et al.* already reported that a brief exposure of oxygen (O_2_) to the growth substrate is mandatory in the initial phase of synthesis to etch the carbon atoms introduced by iodixanol, which the authors added to their Mo-solution to improve adhesion to the substrate during spin-coating, and subsequently to promote the formation of large MoS_2_ flakes.^34^ Even in the absence of an extra carbon source, such as iodixanol, we found that O_2_ has a strong effect on the morphology of MoS_2_, while synthesis of triangular shaped MoS_2_ single layer flakes is impossible in the absence of O_2_ (Fig. 2a and Fig. S31†). Yet, O_2_ exposure throughout the synthesis must be avoided, as MoS_2_ is susceptible to oxidation (Fig. 2b).^36,37^ To determine the impact of O_2_ exposure time on the formation and oxidation of MoS_2_ flakes, we performed X-ray photoelectron spectroscopy (XPS) on a series of samples synthesized at 710 °C for 6 min with different exposure times of O_2_ (1.3% in nitrogen, see Experimental and Table S1†). Analysis of the high-resolution X-ray photoelectron (XP) spectra reveals the disappearance of the Mo 3d signal of MoS_2_ and the concurrent rise of a Mo^6+^ signal of oxidized molybdenum with increasing exposure time of O_2_ (Fig. 2c). Oxidation of MoS_2_ is also confirmed by the disappearance of S 2s and S 2p signals and the concurrent rise of a sulfate signal. The apparent S/Mo-ratio, determined by the formula described in Experimental, continuously decreases with exposure time of O_2_ longer than 10 min, further corroborating the oxidation of MoS_2_ flakes (Fig. S32†). The apparent S/Mo-ratio of the sample synthesized without O_2_ in the gas flow is about 1.9, *i.e.*, close to the stoichiometric value of MoS_2_. In addition, the high-resolution XP-spectra of such a sample (Fig. S21†) is readily comparable with those of MoS_2_ powder (Fig. S8†) as well as with those of samples exhibiting large triangular shaped flakes (Fig. S15,† as an example). MoS_2_ thus forms even in absence of O_2_, but the latter has a dramatic impact on the morphology of the product. Based on these findings, we propose that O_2_ burns organic contaminants on the surface of the growth substrate at the beginning of synthesis, enabling lateral growth of MoS_2_ single layer flakes, just as oxygen exposure in a plasma environment serves to remove organic contaminants from surfaces.^38^ However, as the synthesis time progresses, O_2_ becomes detrimental and even has a destructive effect by oxidizing grown MoS_2_ flakes, so the presence of oxygen in the gas phase late in the synthesis is not conducive.

In summary, O_2_ proves to be a crucial parameter in this type of CVD process and must be carefully adjusted to promote the growth of MoS_2_ single layer flakes while avoiding any detrimental oxidation. Unless otherwise specified, in the following experiments, the exposure time of O_2_ is kept constant at about 2.5 min (until the tube furnace reaches a temperature of 200 °C) to determine the impact of temperature and synthesis time on the formation of MoS_2_.

### Formation of MoS_2_ single layer flakes

**Fig. 3:**
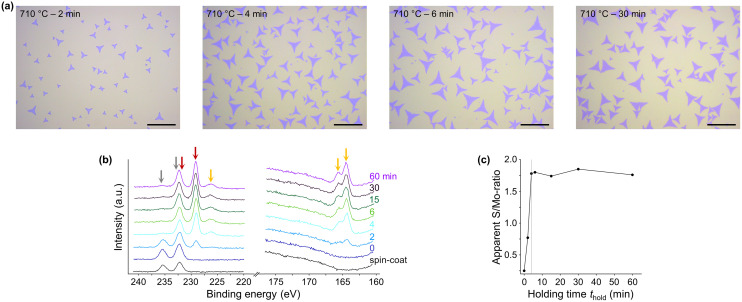
(a) Light microscopy images of samples synthesized at 710 °C for 2, 4, 6, and 30 min, respectively. The scale bar represents 20 μm. (b) High-resolution XP-spectra of Mo 3d, S 2s, and S 2p core level peak regions of samples synthesized at 710 °C and various *t*_hold_. The grey arrows indicate the Mo^6+^-signals, red the Mo^4+^-signals, and orange the sulfur signals of MoS_2_, respectively. (c) Apparent S/Mo-ratio of the samples synthesized at 710 °C with different *t*_hold_, determined by the formula described in the Experimental section. The grey line indicates the *t*_hold_ of 4 min after which no change in the apparent S/Mo-ratio was detected anymore, settling to a value of 1.8 (1.75 ± 0.04), rather close to the expected stoichiometry.

Light microscopy of MoS_2_ flakes synthesized at 710 °C and *t*_hold_ > 4 min shows no discernible differences in size and number with respect to *t*_hold_, suggesting a short time frame required for the synthesis of MoS_2_ flakes (Fig. 3a). Analysis of the high-resolution XP-spectra of samples synthesized at 710 °C and various *t*_hold_ confirms the formation of MoS_2_ by the disappearance of the Mo 3d signal of Na_2_MoO_4_ and the concurrent rise of that of MoS_2_ with increasing *t*_hold_, as well as the appearance of S 2s and S 2p signals (Fig. 3b and additional samples in ESI† section XPS analysis). Since no significant changes in the apparent S/Mo-ratio were measured for all samples with *t*_hold_ > 4 min, the conversion of Na_2_MoO_4_ to MoS_2_ was found to be completed at very short *t*_hold_ (Fig. 3c). The O 1s signal of Na_2_MoO_4_ also becomes undetectable for all samples with a *t*_hold_ > 4 min, corroborating the fast consumption of the Mo-source and conversion to MoS_2_ (Fig. S33†). However, a minor peak at the binding energy range characteristic of Mo^6+^ 3d_3/2_ remained observable even at the highest *t*_hold_. This spectral feature might indicate the presence of unreacted molybdate, or the occurrence of some minor re-oxidation of MoS_2_ flakes after exposure to air. Alternatively, the signal could entirely constitute the secondary structure of the Mo 3d signal of MoS_2_, as found by Wang *et al.* for the case of nearly pure MoS_2_.^39^ In our samples, small amounts of oxidized MoS_2_ cannot be unambiguously identified by the analysis of the O 1s region, as the latter is dominated by the signal of the SiO_2_/Si substrate, which partially overlaps with the O 1s signal of oxidized MoS_2_ (Fig. S33†). Despite the curve fitting model used in this work assumes that the minor peak at the binding energy range characteristic of Mo 3d_3/2_ of oxidized MoS_2_ is solely ascribed to such species, the actual interpretation of the signal may be not so straightforward.

Overall, the XPS analysis reveals that MoS_2_ flakes can be synthesized within 2 min at 710 °C and extending beyond 4 min does not affect their size, number, and chemistry. In the following, we explore a range of *T*_hold_ (710–810 °C) and *t*_hold_ (2–60 min) and study their influence on the MoS_2_ flakes.

### Influence of temperature–time-profile

The temperature–time-profile (*T*–*t*-profile) is defined by the maximum temperature (holding temperature, *T*_hold_) and the duration of *T*_hold_ (holding time, *t*_hold_) and applies to the center of the tube furnace, where the tube furnace thermocouple and growth substrate were placed. Since the synthesis and any other reactions can also occur outside the *t*_hold_ timeframe during heating and cooling, we introduced an additional parameter denoted as synthesis time (*t*_syn_), indicating the time during which the temperature in the center of the tube furnace is higher than 650 °C (see Experimental). By using different *T*–*t*-profiles, other synthesis parameters, such as the sulfur (S) content in the gas phase, which is governed by the temperature in the S-evaporating zone (see Experimental), will also be affected. To exclusively investigate the influence of *T*–*t*-profile, a single synthesis was conducted at 790 °C for 10 min, using 14 growth substrates placed in a row within the reaction tube (14-sample test). Taking advantage of the temperature gradient within the tube furnace, *T*_hold_ and *t*_syn_ increase and decrease progressively for the growth substrates along the reaction tube, reaching a maximum for those situated in the center, the standard synthesis position. This allows simultaneous synthesis of samples with different *T*–*t*-profiles, while keeping the sulfur content as constant as possible. The growth substrates were labeled from I to XIV, starting with the sample located in the upstream heating zone, and further described by their distance from the center of the tube furnace, the position of growth substrate number VII (Fig. S3†). We always refer to these samples by the term “14-sample test” to distinguish them from the results obtained by the standard synthesis conducted with a single growth substrate positioned at the center of the tube furnace.

**Fig. 4:**
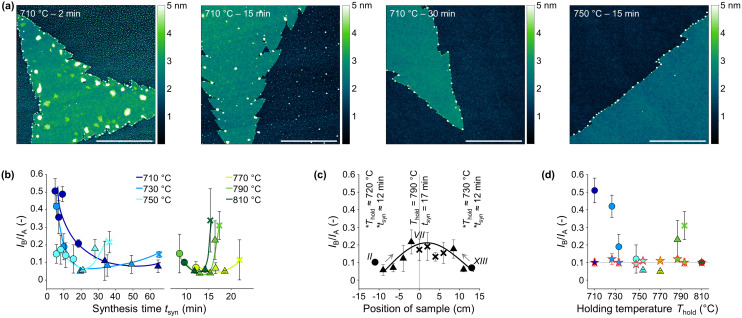
(a) AFM measurements of samples synthesized with different *T*–*t*-profiles. The scale bars represent 2 μm. (b)–(d) The shape of the markers indicate the degree of detachability, where circles represent 100% detachability, triangles represent partial detachability with varying degrees, and crosses no detachability at all (see Fig. 5). (b) *I*_B_/*I*_A_-ratio *versus* synthesis time *t*_syn_ of all synthesized samples listed in ESI Table S2,† where different colors represent the corresponding *T*_hold_. Low *I*_B_/*I*_A_-ratio indicates low density of defects and high crystal quality.^46^ (c) *I*_B_/*I*_A_-ratio of the samples synthesized in the 14-sample test. The grey vertical line indicates the center of the tube furnace, where sample number VII was placed. The samples I and XIV were excluded, as they did not pass the first quality screening with the light microscope (see Fig. S35†). (d) *I*_B_/*I*_A_-ratio of various samples before (symbols and colors as in b) and after transfer (stars) with the PMMA-assisted transfer process. All measurements are conducted after a storage time of at least 48 hours, after which no further water adsorption is expected, influencing the measurement.

For the holding temperatures and times tested (710–810 °C and 4–60 min, respectively), light microscopy shows no obvious effects on the morphology of the MoS_2_ flakes, which also applies to flakes synthesized in the 14-sample test (examples shown in Fig. S34 and S35†). All flakes are triangular in shape, evenly distributed over the complete growth substrate and similar in size and number independent on the applied *T*–*t*-profile. Furthermore, all flakes show the same color indicating an identical flake thickness,^40,41^ except for the most upstream and most downstream samples placed in the tube furnace in the 14-sample test (samples I and XIV in S35†). Measurements with an external thermocouple indicate that *T*_hold_ was about 660 °C at the position of growth substrate number XIV, suggesting unfavorable process conditions. Therefore, samples I and XIV are excluded from further analysis. It is worth noting that the synthesized MoS_2_ flakes don't exhibit exact triangular shapes but have slightly concave edges. Despite the former has lowest formation energy, various morphologies such as truncated triangles, hexagons, and dendritic (snowflakes) can easily be synthesized with CVD, by varying the growth environment, such as holding temperatures of precursors, position of growth substrate, and synthesis time.^24,42,43^ In the end, the molybdenum to sulfur (Mo_source_/S_source_) ratio, which can be tuned by the as mentioned synthesis parameters, determines the shape of MoS_2_ flakes. In our synthesis method, we assume a similar growth environment during the formation of MoS_2_ flakes for each *T*–*t*-profile investigated, resulting in the same shape of flakes, as first the amount of molybdenum available for the synthesis is constant for each synthesis. Second, each *T*–*t*-profile studied had the same heating rate, and the sulfur crucible was placed so that the sulfur melted at the same time during heating, resulting in the same Mo_source_/S_source_-ratio within the short time frame the MoS_2_ flakes are formed. The Raman spectra of all samples show two characteristic peaks at around 404 cm^−1^ and 384 cm^−1^ representing the out-of-plane vibration mode A_1g_ and the in-plane vibration mode E_2g_^1^, respectively (Fig. S36†). The difference of about 20 cm^−1^ between the two peaks indicates the presence of single layers (Table S5†).^44,45^ AFM height analysis also results in about 1 nm thickness, thus confirming the presence of single layers (Fig. S37†).^45,46^ Additionally, AFM measurements reveal prominent particle-like features around and on the flakes. The density of these features decreases with increasing *T*_hold_ and *t*_hold_ (Fig. 4a). Flakes exposed to water no longer show such particle-like features and, moreover, no detrimental defects, indicating that these features are not defective structures of the MoS_2_ flakes but rather a water-soluble by-product of the synthesis that is apparently not assimilated into the MoS_2_ flakes (Fig. S38 and S44†). Due to the fast consumption of the Mo-source (see Fig. 3b and c), this by-product is no leftover of the Mo-salt. This by-product raises no concerns as it is not incorporated into the MoS_2_ flakes and can be easily removed through water, a process that naturally occurs during transfer of MoS_2_ flakes using a water-based method. A discussion on the decrease in concentration of the by-product with increasing *T*_hold_ and *t*_hold_ is provided in Role of sodium.

The crystal quality of MoS_2_ flakes was also investigated by photoluminescence (PL). The PL spectrum of a MoS_2_ single layer flake shows a prominent peak at around 1.85 eV and a less dominant one at around 2.0 eV (Fig. S39†). These two resonances are the direct excitonic transitions at the Brillouin zone K point due to the spin–orbit splitting of the valence band and are known as A- and B-exciton, respectively.^47,48^ The ratio of the A- and B-exciton PL intensity (*I*_B_/*I*_A_-ratio) reflects the crystal quality of the MoS_2_ flakes, with a low *I*_B_/*I*_A_-ratio indicating a low density of defects and high sample quality.^49^ When analyzed on the growth substrate, flakes synthesized with different *T*–*t*-profiles strongly differ in their measured *I*_B_/*I*_A_-ratios, showing a parabolic trend with increasing *t*_syn_ for each *T*_hold_ (Fig. 4b). The *I*_B_/*I*_A_-ratio of the samples synthesized in the 14-sample test exhibits an increasing trend the closer the sample was placed to the center of the tube furnace, where it is exposed to a higher *T*_hold_ and longer *t*_syn_ (Fig. 4c). This behavior correlates well with the respective right branches of the parabolas in Fig. 4b, where increasing *t*_syn_ leads to a higher *I*_B_/*I*_A_-ratio. The left branches in Fig. 4b are not discernible in Fig. 4c due to the too high *T*_hold_ and long *t*_hold_ in the 14-sample test. However, any PL measurement of MoS_2_ single layer flakes strongly depends on the substrate material and the interactions the flakes establish with their surroundings.^50–52^ To determine whether the applied *T*–*t*-profile affects the crystal quality or alters the growth substrate–flake interactions, which in turn influence the PL, random samples were transferred onto freshly cleaned SiO_2_/Si substrates using the poly(methyl methacrylate) (PMMA) assisted method (see Experimental). All transferred flakes have a similar *I*_B_/*I*_A_-ratio of 0.1, regardless of the *T*–*t*-profile applied for their synthesis (red stars in Fig. 4d), indicating an equally high quality of the synthesized MoS_2_ flakes. Therefore, the observed differences in PL for MoS_2_ flakes on the growth substrate infer different interactions between the flakes and the substrate, potentially resulting from variations in the concentrations of the by-product, which are dependent on the applied *T*–*t*-profile and will be discussed in more detail later.

**Fig. 5:**
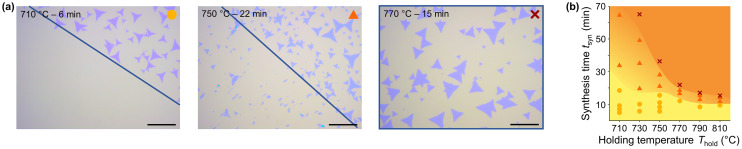
(a) Light microscopy images of three samples synthesized with the *T*–*t*-profile as indicated in the images. The blue line marks the immersion depth of the growth substrate into water to detach the flakes. The sample synthesized at 770 °C for 15 min was completely immersed into water. The scale bar represents 20 μm. (b) Detachability of MoS_2_ flakes as a function of *T*_hold_ and *t*_syn_. The color and shape of the markers indicate the degree of detachability, where yellow circles represent 100% detachability, orange triangles represent partial detachability with varying degree, and red crosses no detachability at all.

For a range of *T*–*t*-profiles, we observed that synthesized MoS_2_ flakes detach from the growth substrate when immersed in water directly after synthesis (Fig. 5a and Fig. S40, S41†). The detached flakes float on the water surface as a greenish carpet and can be collected by immersing a fresh substrate and lifting them off (Fig. S42†). To determine the influence of the *T*–*t*-profile on the detachability, samples were synthesized with different combinations of *T*_hold_ and *t*_hold_ and analyzed by light microscopy after partial immersion of the growth substrate into water. The detachability was subsequently evaluated qualitatively using a rating system with yellow circles representing full (100%) detachability, orange triangles partial detachability, and red crosses no detachability at all (Fig. 5a). The *T*–*t*-profile strongly influences detachability, which generally decreases with increasing *T*_hold_ and *t*_syn_ (Fig. 5b). The transition from full to zero detachability occurs over a wide range of *t*_syn_ for low *T*_hold_ (710–750 °C) and becomes significantly narrower for higher *T*_hold_. The same trend is evident for *t*_syn_, where the detachability decreases rapidly with increasing *T*_hold_ for long *t*_syn_. The samples synthesized in the 14-sample test show a reduction in detachability for the samples placed closer to the center of the tube furnace, where they are exposed to a higher *T*_hold_ and longer *t*_syn_ (Fig. 4c and S41†). This finding corroborates the relationship between the *T*–*t*-profile and detachability as it excludes other parameters influenced by the *T*–*t*-profile that could potentially account for the variation in detachability. Comparison of the *I*_B_/*I*_A_-ratio measured by PL on the growth substrate with the degree of detachability shows for all samples, including those synthesized in the 14-sample test, that the *I*_B_/*I*_A_-ratio minimum is always around the *t*_syn_ at which the samples start to lose their detachability (Fig. 4b and c). Further, the observed parabola compresses with increasing *T*_hold_ (Fig. 4b), just as the transition range between water-detachable and non-detachable decreases. This correlation additionally illustrates the influence of the *T*–*t*-profile on the growth substrate–flake interaction and suggests that the observed influence of the *T*–*t*-profile on PL and detachability is likely attributable to the same underlying cause.

Besides the *T*–*t*-profile, storage of MoS_2_ flakes in ambient conditions also affects detachability, becoming lower with increasing storage time. Notably, in dry environments, this effect is suppressed, indicating a direct involvement of humidity on the aging process (Fig. S43†). The formation of particle-like features and the *I*_B_/*I*_A_-ratio measured by PL also depend on the storage time. Samples measured by AFM directly after synthesis show a smooth surface without any significant features, which appear only after about 12 hours of storage in ambient conditions (22 °C, 40–60% RH, Fig. S44†). The *I*_B_/*I*_A_-ratio of samples with water-detachable flakes strongly changes in the first 40 hours of storage, while samples with non-detachable flakes have a constant *I*_B_/*I*_A_-ratio (Fig. S45†). These findings confirm that the by-product governs growth substrate–flake interactions, consequently being responsible for variations in PL and detachability. However, the parabolic behavior of the PL measurements cannot be rationalized at this point yet.

In summary, the *T*–*t*-profiles tested show no influence on shape, size, number of layers, and quality of the MoS_2_ flakes, suggesting a wide range of suitable *T*–*t*-profiles for simple and fast synthesis of MoS_2_ single layer flakes in high quality and large quantity. The XPS and AFM measurements unveil the presence of a water-soluble by-product, of which the concentration decreases with increasing *T*_hold_ and *t*_hold_ and thus depends on the *T*–*t*-profile. This by-product, its variation in concentration, and formation of clumps with storage time due to moisture adsorption, are responsible for differences in the growth substrate–flake interaction and thus for the degree of detachability and variances in PL. We expect that this by-product or rather the different growth substrate–flake interaction will also affect the measurements of electrical properties. However, we defer this characterization as it is beyond the scope of this manuscript. Here we investigate the CVD process in more detail by discussing the role of sodium in the synthesis, as we suspect the presence of a sodium-containing by-product.

### Role of sodium

**Fig. 6:**
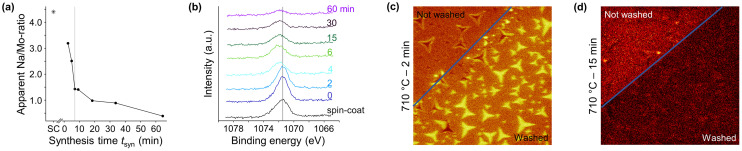
(a) Apparent Na/Mo-ratio of samples synthesized at 710 °C with different *t*_hold_, determined by the formula described in the Experimental. The grey line marks the *t*_hold_ of 4 min after which no changes in the apparent S/Mo-ratio were detected anymore (see Fig. 3b and c). SC marks the sample with spin-coated Na_2_MoO_4_ as a reference. (b) High-resolution XP-spectra of Na 1s signal. (c and d) ToF-SIMS elemental maps of N^+^-ions at a partly washed area of samples synthesized with different *T*–*t*-profiles. The blue lines mark the immersion depth of the growth substrate into water to detach the flakes. The scan size was 100 μm.

Sodium (Na) is known to support the lateral growth of MoS_2_ flakes and is typically added directly to the Mo-source as sodium chloride (NaCl) or sodium hydroxide (NaOH).^34,53^ Since our Mo-source (Na_2_MoO_4_) already contains Na no additional Na was added. Although the XPS measurements reveal that the conversion of Na_2_MoO_4_ to MoS_2_ is completed after a *t*_hold_ of about 4 min (see Fig. 3b and c), the apparent Na/Mo-ratio decreases continuously with increasing *t*_hold_. Moreover, the Na 1s signal shifts towards higher binding energy, indicating chemical changes that still take place after synthesis of MoS_2_ (Fig. 6a and b). If no volatile Na-containing products are formed, the decrease of the apparent Na/Mo-ratio and chemical shift with longer *t*_hold_ indicate a migration of Na into the growth substrate,^54^ suggesting the involvement of the SiO_2_/Si growth substrates in the synthesis process. It is worth noting that the apparent Na/Mo-ratio of spin-coated Na_2_MoO_4_ of 4.4, as measured by XPS, is substantially larger than the expected stoichiometry for Na_2_MoO_4_. The observed deviation is discussed in some detail in the ESI (see ESI† section XPS analysis 2.6). While XPS is a powerful tool to quantitatively analyze the chemical composition of samples, it lacks lateral resolution. Therefore, time-of-flight secondary ion mass spectrometry (ToF-SIMS) measurements were performed to obtain a chemical mapping of the MoS_2_ flakes and growth substrate to identify possible local features and anomalies. No remarkable variances could be detected in the mapping of S^−^- and Mo^+^-ions of samples synthesized with different *T*–*t*-profiles (Fig. S46 and S47†). In contrast, a significant change in the mapping of Na^+^-ions is observed for samples synthesized at varying *T*_hold_ and *t*_hold._ Specifically, a rapid decrease in Na intensity is firstly observed as *T*_hold_ and *t*_hold_ increases, which is in line with the outcomes of the XPS quantitative analysis (Fig. 6c, d and Fig. S48, S49†). Secondly, the areas of the growth substrate that were immersed into water to detach the MoS_2_ flakes show an increased concentration of Na^+^-ions at the sites where MoS_2_ flakes were previously grown (Fig. 6c). These “ghost-flakes” indicate that Na preferentially accumulates between the MoS_2_ flakes and the growth substrate. Finally, in areas where no MoS_2_ flakes were grown, a decrease of Na^+^-ion concentration can be detected in the water-exposed part compared to the non-exposed part (Fig. 6d). These observations indicate the presence of a Na-containing water-soluble by-product and point to a potential key role of Na, not only in supporting the lateral growth of MoS_2_ flakes, but also in mediating the ability to detach the MoS_2_ flakes with water. Possible Na-containing water-soluble by-products formed during the conversion of Na_2_MoO_4_ to MoS_2_ could be sodium sulfite (Na_2_SO_3_) and its in air easily weathered and oxidized form sodium sulfate (Na_2_SO_4_),^55^ or sodium oxide (Na_2_O),^34^ which forms sodium hydrogencarbonate (NaHCO_3_) and sodium carbonate (Na_2_CO_3_) if exposed to air. However, none of these species could be clearly identified in the ToF-SIMS and XPS measurements. Nonetheless, Na_2_O has a temperature-dependent reactivity with SiO_2_ forming sodium silicate glasses,^35^ and can migrate into the substrate (see Fig. 1), diluting in the silica glass matrix, consistent with the decrease in concentration and shift in binding energy of Na with increasing *T*_hold_ and *t*_hold_ as measured by XPS.

To search for possible interactions between Na_2_MoO_4_, SiO_2_, and O_2_ in the gas flow, selected syntheses were repeated in the absence of sulfur (Table S1†). No significant differences were observed in the high-resolution XP-spectra of either the Mo 3d or Na 1s core level peak regions, regardless of the exposure time of O_2_ (Fig. S50†). Thus, when simply heating Na_2_MoO_4_ on SiO_2_/Si, with or without O_2_ present in the gas flow, no chemical reactions take place and only minor amounts of Mo and Na are lost by evaporation or migration into the substrate, despite increased mobility due to Na_2_MoO_4_ being in the liquid state. The reaction of S with Na_2_MoO_4_ to form MoS_2_ is indispensable for the migration of Na into the substrate, strengthening the assumption that a Na-containing by-product with enhanced mobility in SiO_2_ such as Na_2_O, is formed.^34,35^ These findings reveal an involvement of the SiO_2_ in the synthesis of MoS_2_ flakes, and we strongly recommend transferring the synthesized MoS_2_ flakes onto a fresh substrate for further processing, such as device fabrication and characterization, as the growth substrate and growth substrate–flake interaction are greatly affected by the synthesis by-products and substrate modifications through Na migration. Water-based transfer methods enable mitigation of these risks.

Concluding this section, our experiments reveal the presence of a water-soluble and Na-containing by-product around, on, and beneath the synthesized MoS_2_ flakes, significantly affecting the behavior of the MoS_2_ flakes. Synthesis at lower *T*_hold_ (710–750 °C) and shorter *t*_hold_ (4–10 min) results in a high concentration of this Na-by-product. Due to the water-solubility of such by-products, the MoS_2_ flakes easily detach from the growth substrate when immersed in water. Moreover, the hygroscopic nature of the Na-by-product facilitates the adsorption of moisture, forming clumps and creating a water-rich environment surrounding the MoS_2_ flakes. With increasing *T*_hold_ or *t*_hold_, the concentration of Na-by-product diminishes due to its migration into the growth substrate. Consequently, the local environment experienced by the MoS_2_ flakes changes, leading to a shift in the *I*_B_/*I*_A_-ratio measured by PL. At certain combinations of *T*_hold_ and *t*_hold_, the MoS_2_ flakes begin to lose their water-detachability as the Na-by-product migrates away from the interphase between the growth substrate and flakes, resulting in a direct growth substrate–flake contact. This unmediated connection apparently inhibits the detachment of MoS_2_ flakes from the growth substrate by water. At this point, the *I*_B_/*I*_A_-ratio has its minimal value (see Fig. 4b). With further increase in *T*_hold_ and *t*_hold_, direct contacts between growth substrate and flake increase, leading to a reduced degree of detachability until it is no longer possible to detach the MoS_2_ flakes at all. The *I*_B_/*I*_A_-ratio increases again, which may be attributed to induced strain due to the lattice mismatch between growth substrate and MoS_2_ flakes, as well as differences in thermal conductivity.^56^ The transferred MoS_2_ flakes are free of any Na-by-product, due to its water solubility and the water-based transfer method used. Furthermore, the flakes are not in direct contact with the fresh substrate, as water clusters are expected to be trapped between the transferred flakes and the fresh substrate. Due to the absence of particles and direct substrate–flake contact a low *I*_B_/*I*_A_-ratio is measured.

## Conclusions

We have presented a simple CVD method to synthesize MoS_2_ single layer flakes that cover the entire growth substrate with high uniformity and good quality, using Na_2_MoO_4_ deposited directly on the growth substrate (SiO_2_/Si) by spin-coating its aqueous solution and sulfur powder as Mo- and S-source, respectively. In-depth studies using Raman, PL, AFM, XPS, and Tof-SIMS measurements firstly showed that the composition of the gas flow must be carefully selected to promote the growth of MoS_2_ single layer flakes and to avoid any detrimental oxidation of the synthesized flakes. Secondly, the study shows that the reaction to form MoS_2_ flakes takes place within the first few minutes of synthesis at temperatures higher than 710 °C and long synthesis time is not necessary. Moreover, the range of suitable *T*–*t*-profiles for the synthesis of MoS_2_ single layer flakes is very wide, rendering this CVD method very robust, insensitive to variations during the synthesis process, and adaptable to different types of tube furnaces. However, the *T*–*t*-profile exhibited a strong influence on the growth substrate–flake interaction and the detachability of MoS_2_ flakes from the growth substrate with water. While no apparent flake parameter correlated with the detachability of the MoS_2_ flakes, the growth substrate–flake interaction was found to be governed by a Na-containing by-product derived from the Mo-source. The presence of this Na-containing by-product, particularly facilitated by low *T*_hold_ and short *t*_hold_, and its water-solubility enables the detachment of MoS_2_ flakes. In contrast, high *T*_hold_ and long *t*_hold_ promote the migration of this Na-by-product into the growth substrate, in turn decreasing its concentration below the MoS_2_ flakes and thus hindering detachability.

## Experimental

### Sample fabrication

#### Materials

Sulfur S (99.98%, CAS: 7704-34-9) and sodium molybdate Na_2_MoO_4_ (≥98%, CAS: 7631-95-0) were purchased from Sigma-Aldrich and used as delivered without any further purification. The aqueous solution of Na_2_MoO_4_ with a concentration of 0.5 mg ml^−1^ was prepared with deionized water. The growth substrate was a silicon (100) n-type wafer (4-inch) with a 300 nm thick thermal oxide layer (SiO_2_/Si). The gases required for synthesis were ultra-pure nitrogen (99.999% N_2_) and an oxygen–nitrogen mixture (10% O_2_ (99.95%) in N_2_ (99.999%)), which were purchased from PanGas.

#### Growth substrate preparation

The growth substrate was cut into pieces of 15 × 15 mm using a wafer dicer (ProVectus LA 7100, Zhengzhou, China), prior to successively cleaning in an ultrasonic bath with acetone, isopropanol, and deionized water for 15 to 20 minutes to remove the polymeric protection layer necessary for cutting. To improve the wettability of SiO_2_ and homogeneous distribution of the Mo-source, the growth substrate was transformed from hydrophobic to hydrophilic using oxygen plasma (600 W for 3 min),^57,58^ prior to spin-coating the aqueous solution of Na_2_MoO_4_ at 4000 rpm for 40 s.

#### Synthesis of MoS_2_

The MoS_2_ flakes were synthesized in a 1-inch single heating zone tube furnace Blue M™ Mini-Mite (Lindberg, Riverside, USA) by enabling the reaction of sulfur vapor and liquidized Na_2_MoO_4_ directly on the growth substrate at atmospheric pressure and at elevated temperatures (ESI† section Synthesis of MoS_2_). In particular, the growth substrate was positioned at the center of the tube furnace directly after spin-coating the aqueous solution of Na_2_MoO_4_. 30 mg sulfur was placed in a small crucible (∅ = 5 mm) at the entrance of the tube furnace in the upstream heating zone. The small crucible confines the sulfur powder and facilitates its precise positioning in the tube, allowing accurate control of the melting time of the sulfur. Right after assembly, the temperature in the tube furnace was gradually increased to a specific temperature (holding temperature, *T*_hold_) within an adjusted ramp-up time of approximately 68 °C min^−1^ for all investigated *T*_hold_. The temperature was kept at *T*_hold_ for a specific time (holding time *t*_hold_), followed by cooling *via* convection down to room temperature (Fig. S2†). To accelerate the cooling and accordingly stop the synthesis the tube furnace was partially opened at a temperature of 650 °C and fully opened at 570 °C. These changes in temperature with time define the temperature–time-profile (*T*–*t*-profile). However chemical reactions do not occur exclusively during *t*_hold_ when the tube furnace is at *T*_hold_ but may start earlier and will continue while the tube furnace cools down. As the cooling takes a finite amount of time, which in turn depends strongly on *T*_hold_, an additional parameter denoted as synthesis time *t*_syn_ was introduced. This *t*_syn_ represents an effective synthesis time and is given by the time during which the temperature in the center of the tube furnace is higher than 650 °C, which ensures that the sulfur source is in liquid state (*i.e.* >120 °C) at its position at the entrance of the tube furnace. We found that insufficient amounts of sulfur are present in the gas phase if only relying on sublimation of powder. Furthermore, diffusion of Mo is low for temperatures lower than 650 °C due to the melting point of Na_2_MoO_4_ at 687 °C. The composition of the gas flow was a combination of ultra-pure nitrogen (pure N_2_) and an oxygen–nitrogen (O_2_/N_2_) mixture (10% O_2_ in N_2_). The pure N_2_ gas ran throughout the synthesis with a flow profile depending on *T*_hold_ and *t*_hold_. At a temperature of 650 °C in the center of the tube furnace, the sulfur starts to melt, and the pure N_2_ gas flow was reduced from 100 to 50 sccm to create a sulfur-rich environment. After *t*_hold_ elapsed, the pure N_2_ gas flow was increased to 500 sccm to support the cooling of the tube furnace. Unless otherwise specified, the O_2_/N_2_ gas ran only during approximately the first 2.5 min of synthesis with 15 sccm, until the tube furnace reached a temperature of 200 °C. Considering the 100 sccm pure N_2_ carrier gas flow, the concentration of O_2_ in the total gas flow equals 1.3%. All experiments conducted are listed in Tables S1 and S2.† To exclude the influence of other parameters, such as the amount of S in the gas phase or gas flow fluctuations, when changing the *T*–*t*-profile, a single synthesis was performed at 790 °C for 10 min but with 14 growth substrates placed simultaneously in a row in the reaction tube (14-sample test, Fig. S3†). Due to the temperature gradient within the tube furnace, *T*_hold_ and *t*_syn_ decrease from the center towards the end of the reaction tube, allowing samples with different *T*–*t*-profiles to be synthesized at the same time. The growth substrates were labelled from I to XIV, starting with the sample placed in the upstream heating zone, and were further described by their distance from the center of the tube furnace, the position of growth substrate number VII.

#### Transfer of MoS_2_ flakes by the pick-up method

To remove any kind of water-soluble by-products and to avoid any influence of the growth substrate on measurements of MoS_2_ flakes, water-detachable MoS_2_ flakes can be transferred onto a fresh substrate by the pick-up method. Therein, the growth substrate is immersed into water directly after synthesis. The MoS_2_ flakes detach from the growth substrate and float as a greenish carpet on the water surface, where they can be picked up by a fresh substrate of any type of material.

#### Transfer of MoS_2_ flakes by the poly(methyl methacrylate) (PMMA) assisted method

To measure photoluminescence without the influence of the growth substrate, MoS_2_ flakes were transferred from the growth substrate onto a bare 300 nm SiO_2_/Si substrate, which was previously cleaned in an ultrasonic bath successively with acetone, isopropanol, and deionized water for 15 to 20 minutes. To this end, poly(methyl methacrylate) (PMMA, 95k) was spin-coated onto the growth substrate at 4000 rpm for 60 s to create a homogeneous thin layer of PMMA (about 300 nm), and cured overnight at ambient conditions. Prior to peeling off the PMMA layer by floating the substrate on a 1 M KOH solution, the edges of the substrate were trimmed to facilitate penetration of the etchant. To remove all etchant residues after peeling, the PMMA layer was washed three times with deionized water by floating on stirred water. After transferring onto a fresh substrate and drying overnight, the PMMA layer was dissolved by immersing the substrate successively in acetone, isopropanol, and deionized water for 1 min for three times. To remove adsorbed water and possible residues of the PMMA layer, the MoS_2_ flakes were irradiated with a green laser (561 nm, 7.6 mW measured at the sample surface) for 1 min before recording the photoluminescence spectrum (Fig. S51†).^50^

### Sample characterization

All samples were measured after storage for at least 48 hours, if not stated otherwise.

Raman and photoluminescence (PL) spectra were recorded in air at ambient conditions by a NT-MDT (Moscow, Russia) Raman system equipped with a green laser (561 nm, 50 mW) and an 100× objective lens (NA = 0.8). The gratings were 150 lines per mm for PL measurements and 1800 lines per mm for Raman measurements. The peak of silica from the substrate was applied as an internal standard for all spectra.

Measurements with the atomic force microscope (AFM) were performed under ambient conditions using a Cypher S Asylum Research AFM (Oxford Instruments, Santa Barbara, USA) in amplitude modulation mode (AM-AFM). The records were analyzed with Gwyddion.

X-ray photoelectron spectroscopy (XPS) was conducted with a PHI Quantera SXM spectrometer (ULVAC-PHI, Chanhassen, USA), operating in constant-analyzer-energy (CAE) mode and equipped with a monochromatic Al Kα X-ray source (1.487 keV). A nominal beam-spot size of 150 μm was used for all spectral acquisitions. High resolution spectra were acquired with a pass energy and an energy step of 55 and 0.1 eV, respectively. For the survey spectra, the pass energy and step size were 280 and 1.0 eV. All spectra were offset by using the O 1s signal of the growth substrate (SiO_2_/Si) as an internal reference (binding energy of 532.5 eV). A detailed description of the XPS chemical state analysis and curve fittings is provided in the ESI (ESI† section XPS analysis). The atomic ratio of the elements of interest *x*_a_ was estimated from the high-resolution X-ray photoelectron spectra with
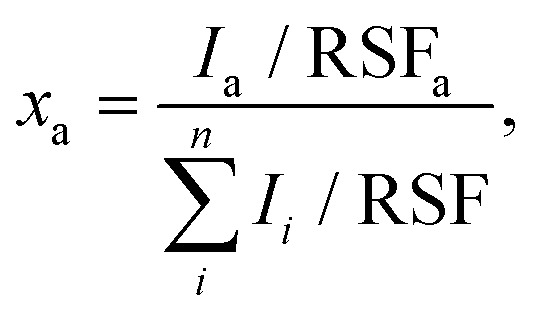
where *I*_*i*_ is the intensity (area) of the peak of element *i* and RSF_*i*_ the associated relative sensitivity factor.^59^ The RSFs used in this work are taken from the spectrometer's software, after correction with the instrument transmission function. It should be noted that the above equation strictly applies to samples with a homogeneous surface material to a depth comparable to the information depth of the analytical method (about 8 nm). As this is clearly not the case for MoS_2_ single layer flakes on SiO_2_, as well as for spin-coated MoS_2_ precursor (aqueous solution of Na_2_MoO_4_) investigated in this work, the “apparent” atomic ratios, *i.e.* the estimates obtained by this equation, may deviate significantly from the actual ratios.

Measurements with the time-of-flight secondary ion mass spectrometry (ToF-SIMS) were performed using an IONTOF, ToF-SIMS 5 instrument (IONTOF, Munster, Germany). A beam of 25 keV Bi^+^ as primary ions was randomly raster-scanned across the area of interest. Images and spectra of the sputtered positive and negative ions were recorded on separate areas of the sample (extraction voltage 2 kV).

## Author contributions

A. Arcifa and R. Kalt conducted the XPS measurements and interpreted the XP-spectra. C. Wäckerlin performed the ToF-SIMS measurements. C. Wäckerlin and R. Kalt analyzed the ToF-SIMS spectra. R. Kalt synthesized all samples and conducted the Raman, PL, and AFM study. R. Kalt devised all experiments and wrote the original draft with input from all authors. A. Stemmer supervised the project, and reviewed, commented, and edited the manuscript drafts.

## Conflicts of interest

There are no conflicts of interest to declare.

## Supplementary Material

NR-015-D3NR03907B-s001
