# Supplementary material for: CVD of MoS2 single layer flakes using Na2MoO4 – impact of oxygen and temperature–time-profile
Source: Nanoscale. 2023 Nov 2;15(46):18871–82. doi: 10.1039/d3nr03907b (PMC10690930; doi:10.1039/d3nr03907b)
Supplement: NR-015-D3NR03907B-s001 [file NR-015-D3NR03907B-s001.pdf]

Electronic Supplementary Material (ESI) for Nanoscale.
This journal is © The Royal Society of Chemistry 2023

Supplementary Information

CVD of MoS_2 Single Layer Flakes using Na_2MoO_4 – Impact of Oxygen and Temperature-Time-Profile

Romana Alice Kalt,[*1] Andrea Arcifa, †[2] Christian Wäckerlin,[3,4] Andreas Stemmer[*1]

[1] Nanotechnology Group, ETH Zurich Säumerstrasse 4, CH-8803 Rüschlikon, Switzerland

[2] Surface Science & Coating Technologies, Swiss Federal Laboratories for Materials Science and Technology (EMPA), Überlandstrasse 129, CH-8600 Dübendorf, Switzerland

[3] Institute of Physics, Swiss Federal Institute of Technology Lausanne (EPFL), Station 3, CH-1015 Lausanne, Switzerland

[4] Laboratory for X-ray Nanoscience and Technologies, Paul-Scherrer-Institute (PSI), CH-5232 Villigen PSI, Switzerland

†Currently at Beyond Gravity Schweiz AG, Schaffhauserstrasse 580, CH-8052 Zürich, Switzerland

[*]Corresponding author

Email: Romana Alice Kalt - kaltro@ethz.ch, Andreas Stemmer - astemmer@ethz.ch

1. Synthesis of MoS_2

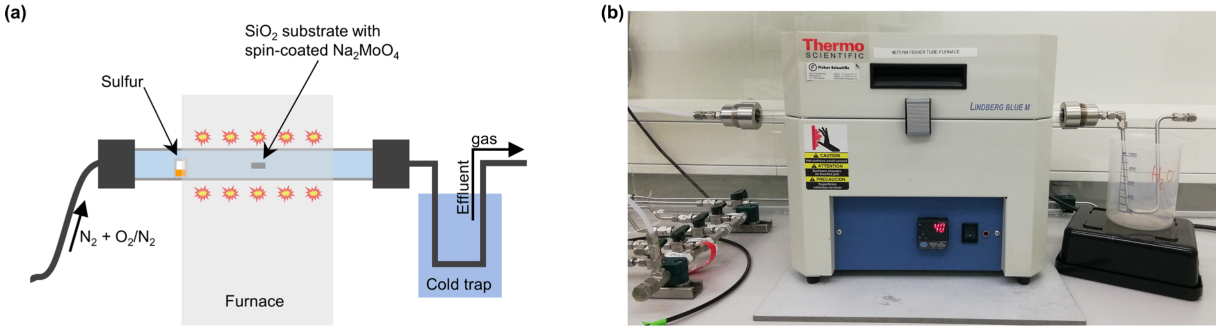

Fig. S1 (a) Schematic drawing of the set-up to synthesize MoS_2 flakes. (b) Picture of the tube furnace.

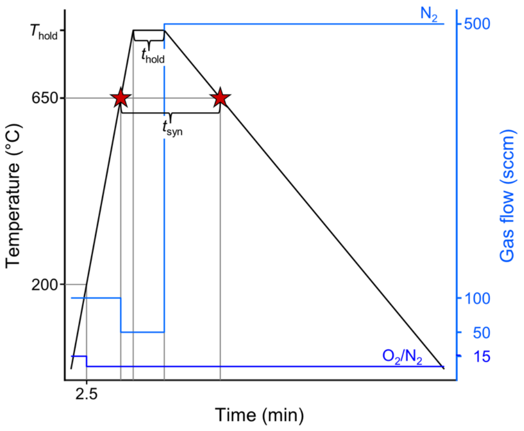

Fig. S2 *T*-*t*-profile (black) and gas flows (blue) during the synthesis of MoS_2 flakes, where light blue represents the pure N_2 and dark blue the O_2/N_2 gas flow. The holding temperature T_{hold} indicates the maximum temperature during synthesis at the center of the tube furnace and the holding time t_{hold} the time the system is at T_{hold}. The synthesis time t_{syn}, which marks the time the center of the tube furnace is at a temperature higher than 650 °C, is flanked by red stars.

Tab. S1 Investigated exposure times of O_2 in the gas flow (1.3 %) and corresponding temperatures in the tube furnace when the O_2/N_2 gas flow was turned off. T_{hold} was 710 °C and t_{hold} was set to 6 min for all exposure times of O_2. The ramp-up time was 10 minutes. The asterisks * indicate the syntheses, which were conducted a second time without sulfur present.

Exposure time of O_2 (min)	Temperature in the tube furnace (°C)
0*	-
2.5*	200
9	650
10*	710
11	710
12	710
13*	710

Tab. S2 Synthesis time t_{syn} for different combinations of T_{hold} and t_{hold} investigated. t_{syn} increases with T_{hold} and t_{hold} due to longer time required for cooling down to 650 °C.

		Holding temperature T_{hold} (°C)					
		710	730	750	770	790	810
Holding time t_{hold} (min)	1	-	-	5.7	-	-	-
	2	4.9	5.8	-	-	8.4	9.5
	3	-	-	8.2	-	-	-
	4	7.0	-	-	-	-	11.7
	6	9.2	10.1	11.1	12.1	12.9	14.3
	7	-	-	-	-	-	15.3
	8	-	-	-	-	15.3	-
	9	-	-	-	-	16.4	-
	10	-	-	15.5	16.5	17.1	-
	12	-	-	-	18.6	-	-
	15	18.4	19.4	21.0	21.9	-	-
	22	-	-	28.0	-	-	-
	30	33.7	35.1	36.3	-	-	-
	45	-	49.1	-	-	-	-
	60	64.5	65.1	-	-	-	-

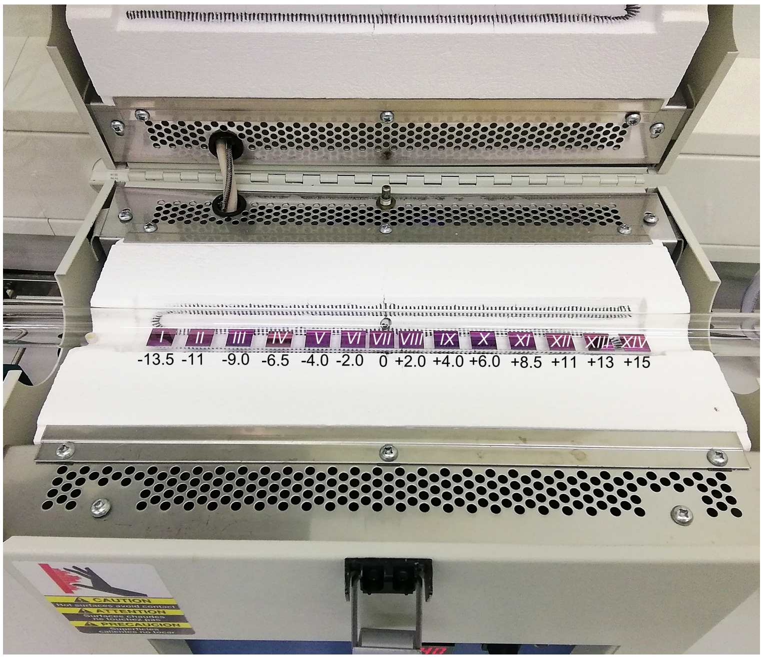

Fig. S3 The 14-sample test was performed by running a synthesis at 790 °C for 10 min with 14 growth substrates placed in a row in the reaction tube to show how the temperature gradient in the tube furnace and therefore the *T*-*t*-profiles influences the reaction process and flake quality.

2. XPS Analysis

2.1 Samples

Tab. S3 Overview of samples investigated with XPS, where T_{hold} denotes the holding temperature, t_{hold} the holding time, and O_{exp} the exposure time of O_2. The parameters in bold represent the parameters of interest.

Sample	Labelling	Description	Remarks
P1	P-MoS2	MoS_2 powder (98 %, CAS: 1317-33-5, from Sigma-Aldrich, as delivered)	Reference sample for binding energy referencing
P2	P-Na2MoO4	Na_2MoO_4 powder (98 %, CAS: 7631-95-0, from Sigma-Aldrich, as delivered)	Control sample for curve fitting model
SC	Na2MoO4	Spin-coated Mo-source (aqueous Na_2MoO_4 solution with 0.5 mg/ml)	Reference sample for Mo-source
Tt0	710C0M	T_{hold} = 710 °C, **t_{hold} = 0 min**, O_{exp} = 2.5 min	
Tt1	710C2M	T_{hold} = 710 °C, **t_{hold} = 2 min**, O_{exp} = 2.5 min	
Tt2	710C4M	T_{hold} = 710 °C, **t_{hold} = 4 min**, O_{exp} = 2.5 min	
Tt3	710C6M	T_{hold} = 710 °C, **t_{hold} = 6 min**, O_{exp} = 2.5 min	
Tt4	710C15M	T_{hold} = 710 °C, **t_{hold} = 15 min**, O_{exp} = 2.5 min	Reference sample for binding energy referencing and curve fitting
Tt5	710C30M	T_{hold} = 710 °C, **t_{hold} = 30 min**, O_{exp} = 2.5 min	
Tt6	710C60M	T_{hold} = 710 °C, **t_{hold} = 60 min**, O_{exp} = 2.5 min	
Tt7	730C15M	**T_{hold} = 730 °C**, **t_{hold} = 15 min**, O_{exp} = 2.5 min	
Tt8	750C15M	**T_{hold} = 750 °C**, **t_{hold} = 15 min**, O_{exp} = 2.5 min	
Tt9	750C30M	**T_{hold} = 750 °C**, **t_{hold} = 30 min**, O_{exp} = 2.5 min	
O0	O0M	T_{hold} = 710 °C, t_{hold} = 6 min, **O_{exp} = 0 min**	
O1	O2.5M	T_{hold} = 710 °C, t_{hold} = 6 min, **O_{exp} = 2.5 min**	Same sample as Tt3
O2	O9M	T_{hold} = 710 °C, t_{hold} = 6 min, **O_{exp} = 9 min**	
O3	O10M	T_{hold} = 710 °C, t_{hold} = 6 min, **O_{exp} = 10 min**	
O4	O11M	T_{hold} = 710 °C, t_{hold} = 6 min, **O_{exp} = 11 min**	
O5	O12M	T_{hold} = 710 °C, t_{hold} = 6 min, **O_{exp} = 12 min**	
O6	O13M	T_{hold} = 710 °C, t_{hold} = 6 min, **O_{exp} = 13 min**	Reference sample for sulfate and curve fitting

S0	S-O0M	T_{hold} = 710 °C, t_{hold} = 6 min, $\boldsymbol{O_{exp}}$ **= 0 min**<br>**No sulfur present**	
S1	S-O2.5M	T_{hold} = 710 °C, t_{hold} = 6 min, $\boldsymbol{O_{exp}}$ **= 2.5 min**<br>**No sulfur present**	
S2	S-O10M	T_{hold} = 710 °C, t_{hold} = 6 min, $\boldsymbol{O_{exp}}$ **= 10 min**<br>**No sulfur present**	
S3	S-O13M	T_{hold} = 710 °C, t_{hold} = 6 min, $\boldsymbol{O_{exp}}$ **= 13 min**<br>**No sulfur present**	

2.2 Survey Spectra

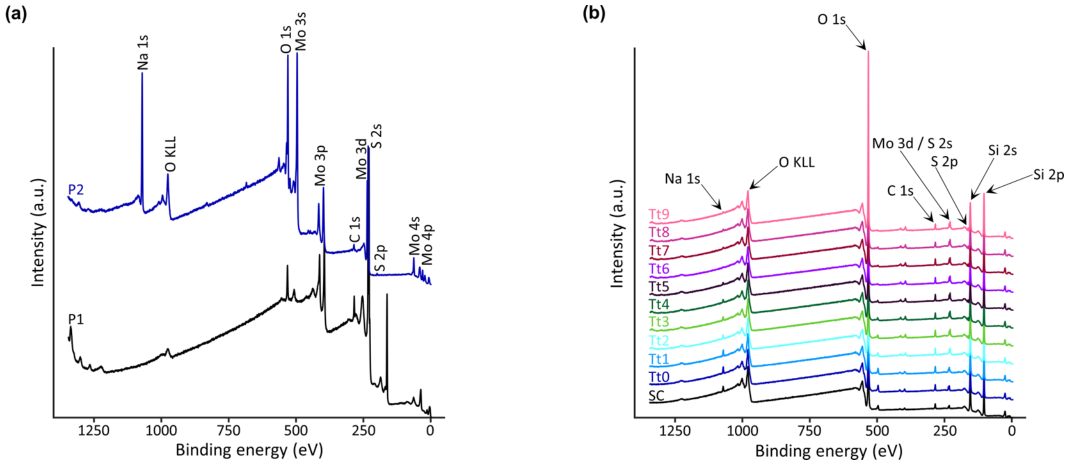

Fig. S4 XP survey spectra of (a) sample P1 (P-MoS2) and P2 (P-Na2MoO4), and (b) MoS_2 flakes synthesized with different temperature-time-profiles as reported in Tab. S3.

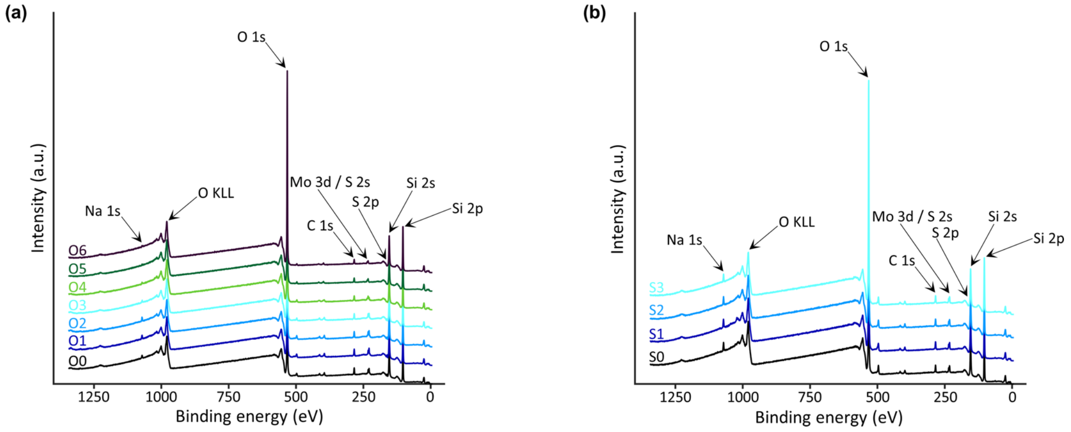

Fig. S5 XP survey spectra of (a) MoS_2 flakes synthesized with different exposure times of O_2 and (b) all samples synthesized without sulfur present as reported in Tab. S1.

2.3 Binding Energy Referencing of High-Resolution XP-Spectra

Due to the insulating nature of the growth substrate used (SiO_2/Si), the following binding energy (BE) referencing method was adopted. First, the XP-spectrum of sample P1 (P-MoS2) was analyzed to obtain a reference spectrum of a conductive sample for the Mo $3d_{5/2}$ peak position of a MoS_2 sample. The peak has its maximum at a binding energy of 229.1 eV and corresponds well to value given in literature.[1,2] Afterwards, the synthesized MoS_2 sample Tt4 (710C15M) was BE-referenced using the Mo 3d peak, set to the value found for P1. The main O 1s of sample Tt4, as for all other synthesized samples, is ascribed to the growth substrate (SiO_2/Si) and was found at a BE of 532.5 eV, corresponding well to literature values.[3] Finally, this O 1s signal of the growth substrate was used for BE referencing of all other synthesized samples.

2.4 Curve Fitting

<u>Background and curve fitting of S 2p</u>

The background of the S 2p signal of MoS_2 has a complex shape as its peaks sit right after the Si 2s signal caused by SiO_2/Si (growth substrate). The effect of the characteristic inelastic scattering losses from the Si 2s signal impacts heavily the S 2p region due to the large difference in material thickness (bulk versus atomically thin layer). To consider the prominent convex shape of the background a quadratic fitting of the background is used. A conventional doublet is used to model the signal of MoS_2 flakes (Fig. S6a), though the signal may actually include a variety of additional minor constituents, such as elemental sulfur, amorphous sulfides (MoS_x with $2<x<3$), partially oxidized MoS_2 (MoS_xO_y), and different MoS_2 phases such as the 2H and 1T phase, which are thus ignored. The signal ascribed to sulfates is fitted with a single conventional doublet too (Fig. S6b), after subtraction of a linear background.

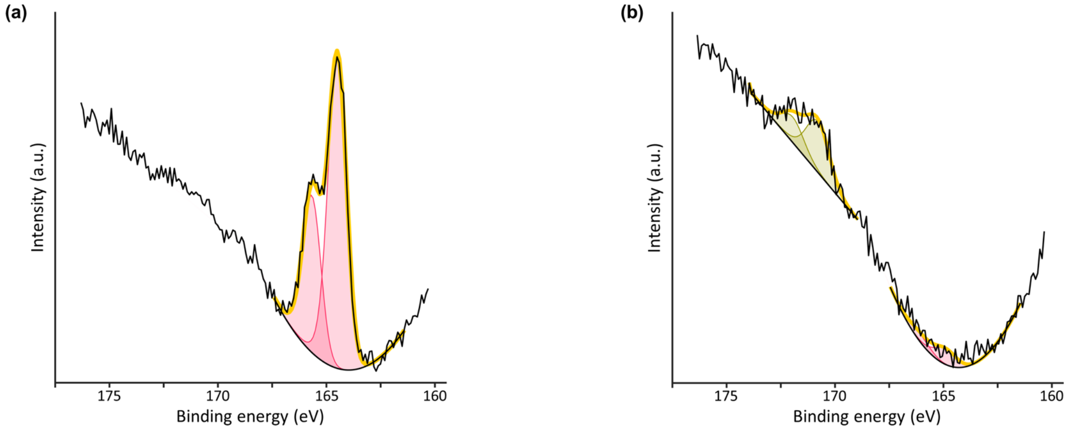

Fig. S6 High-resolution XP-spectra of S 2p core level peak region from the sample (a) Tt4 (710C15M) and (b) O6 (O13M), which are used as reference samples for the curve fitting for the sulfur S 2p doublet (pink) and sulfate (green).

Background and curve fitting of Mo 3d and S 2s

The Mo 3d signals of Na_2MoO_4, MoS_2, and Mo-oxides overlap over a wide BE range, which also encompasses the S 2s signals of MoS_2 and of sulfates, when present.

The following curve-fitting model was applied to represent the signal envelope (Fig. S7).

a.) Singlet and doublet for S 2s and Mo 3d, respectively, representing MoS_2 (pink and purple).

b.) Singlet for S 2s representing sulfates (green). As this peak is entirely overlapped by the Mo 3d envelope, it is not possible to accurately see its presence. Therefore, its curve fitting is only added if a clear S 2p sulfate peak was detected in the high-resolution XP-spectra of the S 2p core level peak region.

c.) Doublet for Mo 3d representing Na_2MoO_4/MoO_3/secondary structure of the MoS_2 signal (grey).

d.) Doublet for Mo_{def} 3d representing defective states such as MoO_{3-x}/MoS_xO_y (dark green).

e.) Shirley background line with two steps at the position of the Mo $3d_{5/2}$ and Mo $3d_{3/2}$ peaks of the MoS_2 signal.

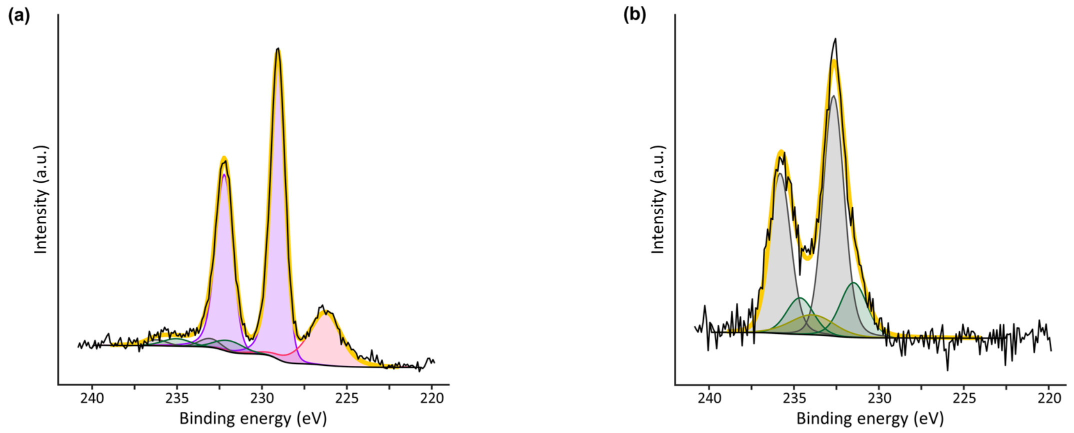

Fig. S7 High-resolution XP-spectra of Mo 3d core level peak region of the sample (a) Tt4 (710C15M) and (b) O6 (O13M), which are used as reference samples for the curve fitting for the sulfur S 2s singlet (pink), Mo 3d doublet (purple), Mo doublet (grey), Mo_{def} doublet (dark green), and sulfate (green).

Overview of fitting parameters

Tab. S4 All parameters and constraints used for the XPS curve fittings.

Signal	Labelling	Source	Background	Position (eV)	Area	FWHM (eV)	Line Shape
Mo $3d_{5/2}$	A_{MoS}	MoS_2	Shirley	228.9 – 229.5	Free	0.9 – 1.1	LA(1.2, 1.6, 260)
Mo $3d_{3/2}$	B_{MoS}	MoS_2	Shirley	A_{MoS} + 3.15[a]	A_{MoS} * 2/3[b]	A_{MoS} * 1.15[a]	LA (1.2, 1.6, 260)
Mo $3d_{5/2}$	A_{Mo6}	Na_2MoO_4, MoO_3, Secondary structure of MoS_2 signal	Shirley	232.3 – 233.1	Free	1.29 – 1.49	LA (1.3, 1.2, 399)
Mo $3d_{3/2}$	B_{Mo6}	Na_2MoO_4, MoO_3, Secondary structure of MoS_2 signal	Shirley	A_{Mo6} + 3.15[a]	A_{Mo6} * 2/3[b]	A_{Mo6} * 1[a]	LA (1.3, 1.2, 399)
Mo $3d_{5/2}$	A_{Modef}	MoO_{3-x}, MoO_xS_y[c]	Shirley	A_{Mo6} – 1.2[d]	Free	A_{Mo6} * 1.28[d]	LA (1.5, 243)
Mo $3d_{3/2}$	B_{Modef}	MoO_{3-x}, MoO_xS_y[c]	Shirley	A_{Modef} + 3.15[a]	A_{Modef} * 2/3[b]	A_{Modef} * 1[a]	LA (1.5, 243)
S 2s	S_{MoS}	MoS_2	Shirley	A_{MoS} – 2.85[e]	A_{MoS} * 0.41[e]	A_{MoS} * 2.11[e]	LA (1.2, 1.6, 100)
S 2s[f]	S_{Sul}	Sulfate	Shirley	233.5 – 234-3	187 – 227[g]	2.6 – 3.1	LA (1.5, 243)
S $2p_{3/2}$	SA_{MoS}	MoS_2	Quadratic	161.5 – 162.3	Free	0.79 – 1.19	LA(1.6, 4, 308)
S $2p_{1/2}$	SB_{MoS}	MoS_2	Quadratic	SA_{MoS} + 1.18[a]	SA_{MoS} * 1[b]	SA_{MoS} * 1[a]	LA(1.6, 4, 308)
S $2p_{3/2}$	SA_{Sul}	Sulfate	Linear	167.9 – 168.7	Free	1.2 – 1.6	LA(1.6,2, 230)
S $2p_{1/2}$	SB_{Sul}	Sulfate	Linear	SA_{Sul} + 1.18[a]	SA_{Sul} * 1[b]	SA_{Sul} * 1[a]	LA(1.5, 243)

[a] These constraints correspond well to values given in literature.[1,2,7]
[b] Area ratio based on degeneracy.
[c] Includes all defective states.
[d] This constraint is derived from the sample O6 (O13M), which shows clearly oxidized MoS_2 flakes.
[e] Assumption that MoS_2 is not structurally changing a lot among samples. Therefore, the relative distance on the binding energy scale, the ratio of area, and fwhm of two atoms of the same constituent must be constant. The values of the constraints are derived from the sample Tt4 (710C15M), which consists mainly of MoS_2 flakes.
[f] This curve fitting is only added if a clear sulfate peak is visible in the high-resolution XP-spectra of the S 2p core level peak region.
[g] As the intensity ratio between 2s and 2p stays the same for each element, the area range of the sulfate peak is derived as +/- 10 % of I_{S2s}^{sul} with $I_{S2s}^{sul} = I_{S2s}^{s}/I_{S2p}^{s} * I_{S2p}^{sul}$ with I representing the intensity of the S 2s and S 2p peak of sulfur s and sulfate sul, respectively.

2.5 High-Resolution XP-Spectra

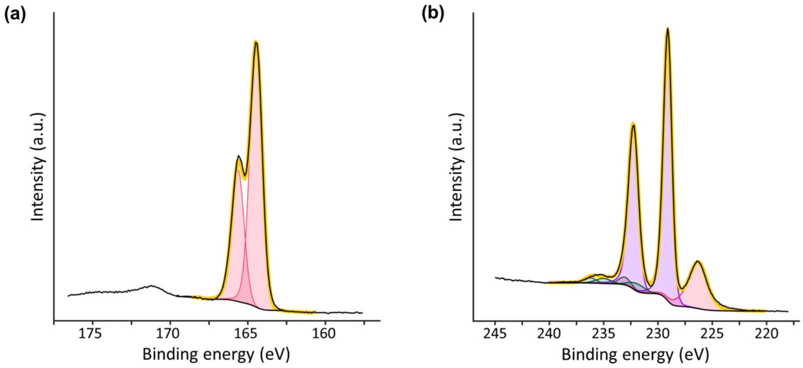

Fig. S8 High-resolution XP-spectra of (a) S 2p and (b) Mo 3d core level peak regions of the sample P1 (P-MoS2).

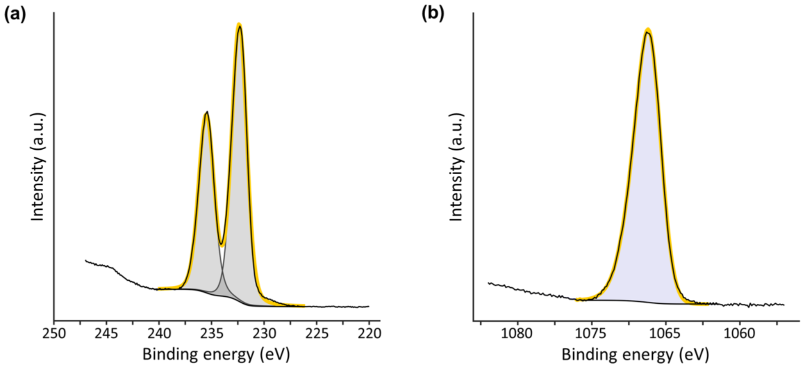

Fig. S9 High-resolution XP-spectra of (a) Mo 3d and (b) Na 1s core level peak regions of the sample P2 (P-Na2MoO4).

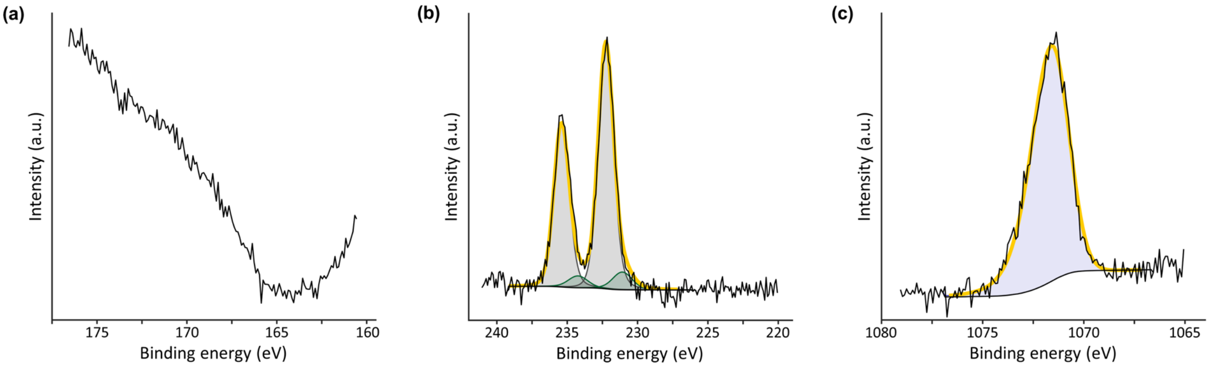

Fig. S10 High-resolution XP-spectra of (a) S 2p, (b) Mo 3d, and (c) Na 1s core level peak regions of the sample SC (Na2MoO4).

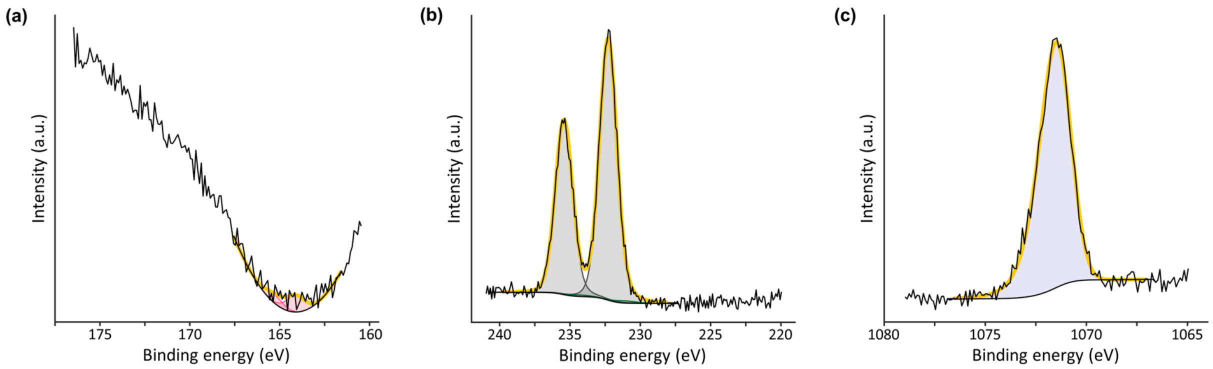

Fig. S11 High-resolution XP-spectra of (a) S 2p, (b) Mo 3d, and (c) Na 1s core level peak regions of the sample Tt0 (710C0M).

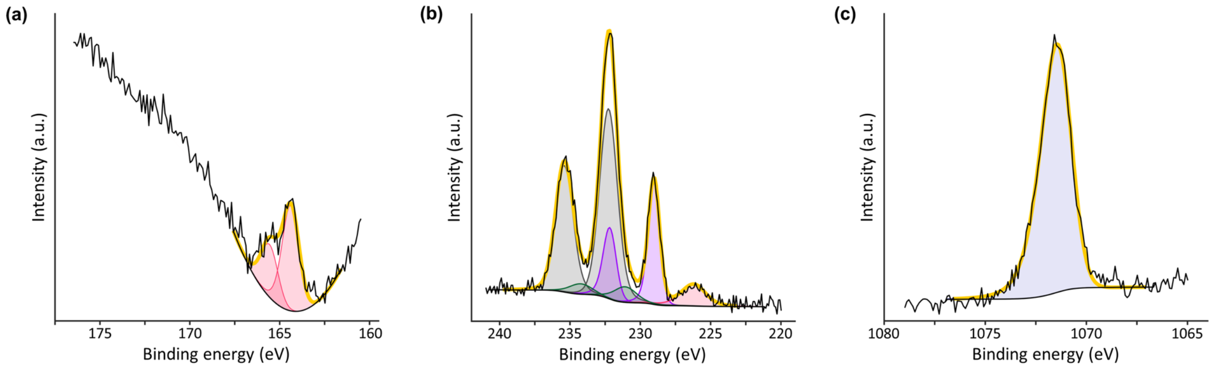

Fig. S12 High-resolution XP-spectra of (a) S 2p, (b) Mo 3d, and (c) Na 1s core level peak regions of the sample Tt1 (710C2M).

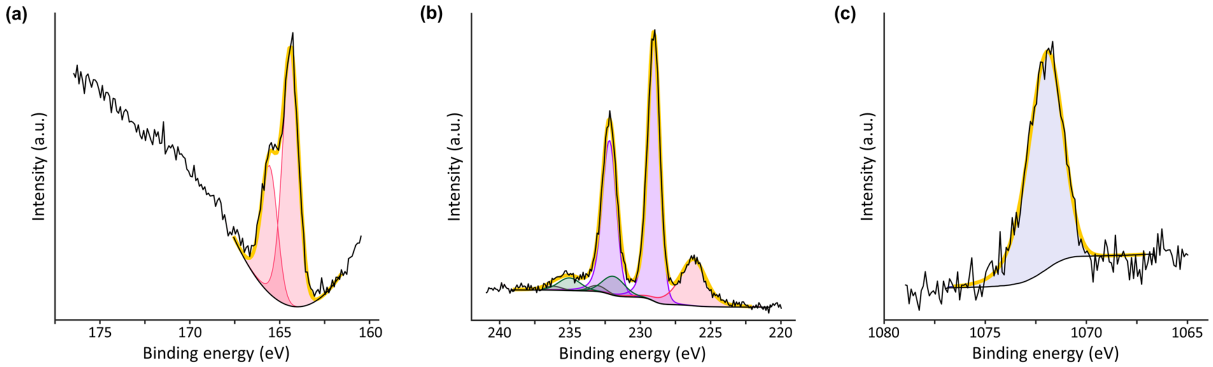

Fig. S13 High-resolution XP-spectra of (a) S 2p, (b) Mo 3d, and (c) Na 1s core level peak regions of the sample Tt2 (710C4M).

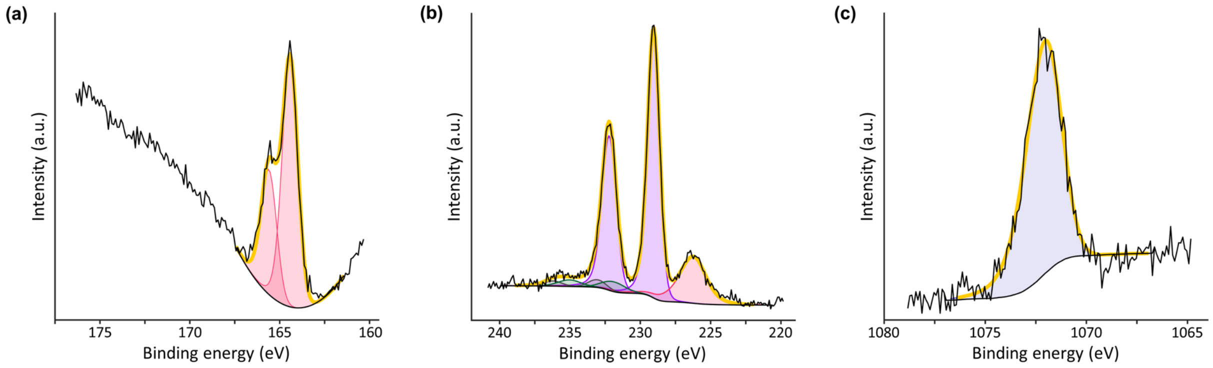

Fig. S14 High-resolution XP-spectra of (a) S 2p, (b) Mo 3d, and (c) Na 1s core level peak regions of the sample Tt3 (710C6M, and O1 (O2.5M), respectively).

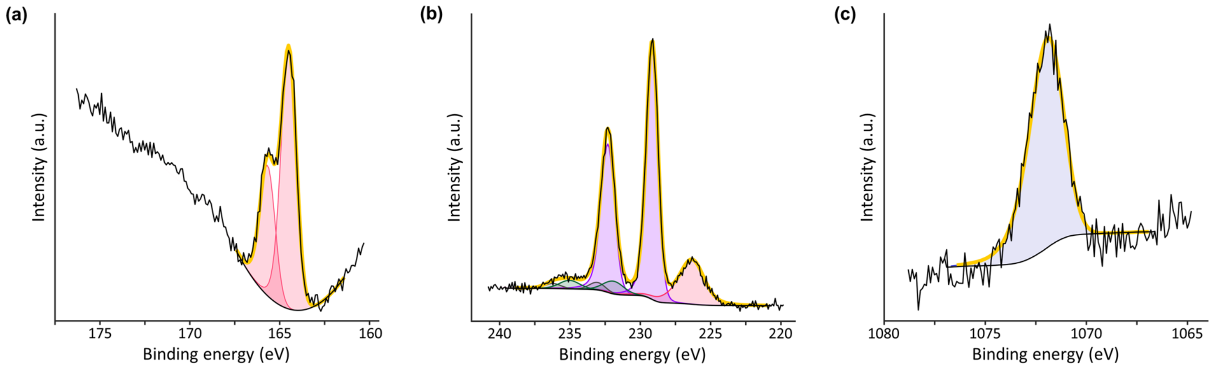

Fig. S15 High-resolution XP-spectra of (a) S 2p, (b) Mo 3d, and (c) Na 1s core level peak regions of the sample Tt4 (710C15M).

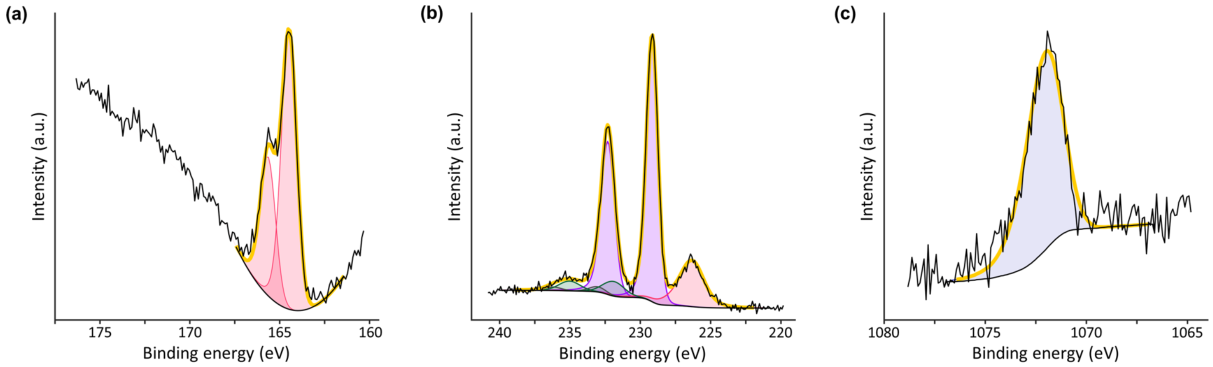

Fig. S17 High-resolution XP-spectra of (a) S 2p, (b) Mo 3d, and (c) Na 1s core level peak regions of the sample Tt5 (710C30M).

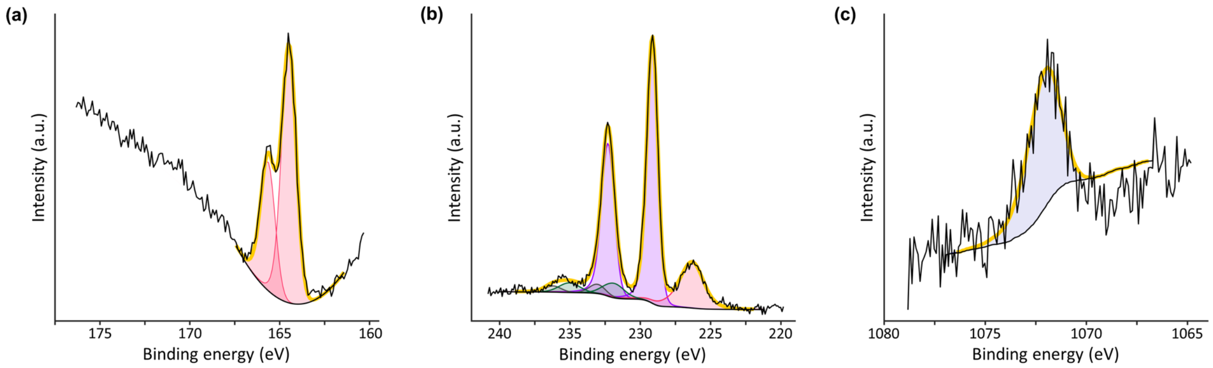

Fig. S16 High-resolution XP-spectra of (a) S 2p, (b) Mo 3d, and (c) Na 1s core level peak regions of the sample Tt6 (710C60M).

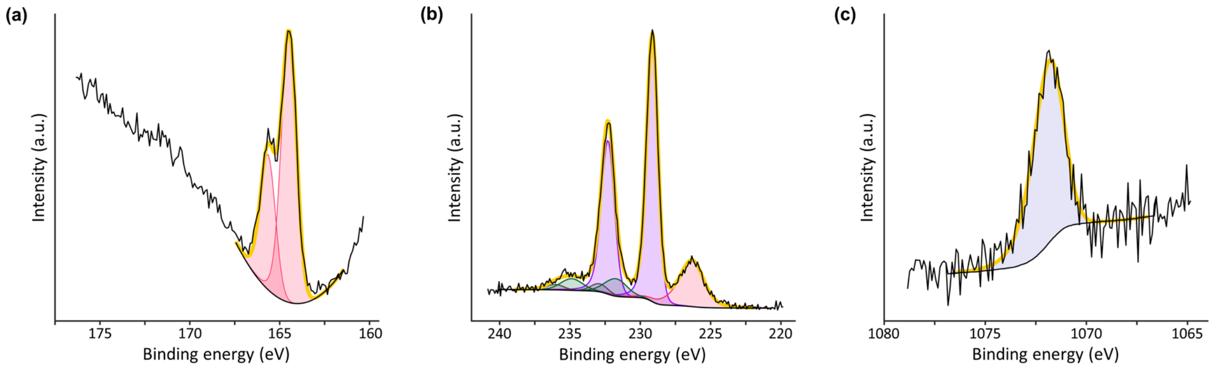

Fig. S18 High-resolution XP-spectra of (a) S 2p, (b) Mo 3d, and (c) Na 1s core level peak regions of the sample Tt7 (730C15M).

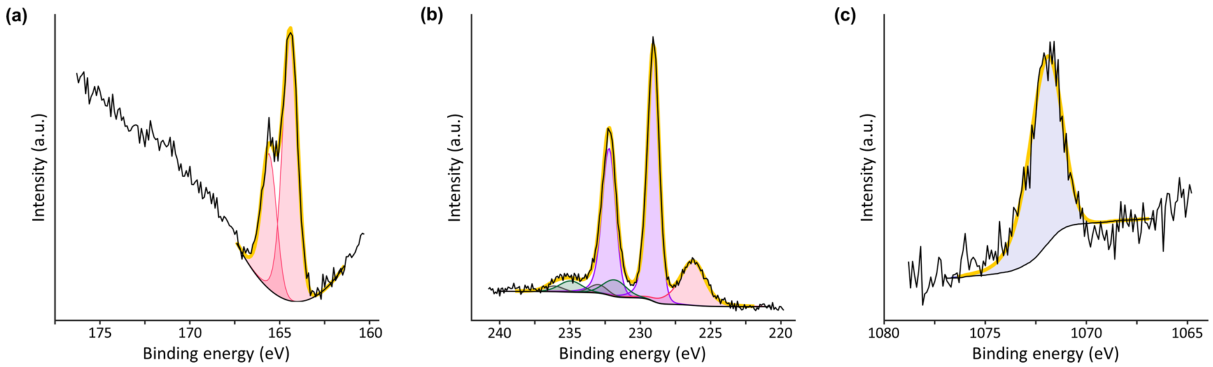

Fig. S19 High-resolution XP-spectra of (a) S 2p, (b) Mo 3d, and (c) Na 1s core level peak regions of the sample Tt8 (750C15M).

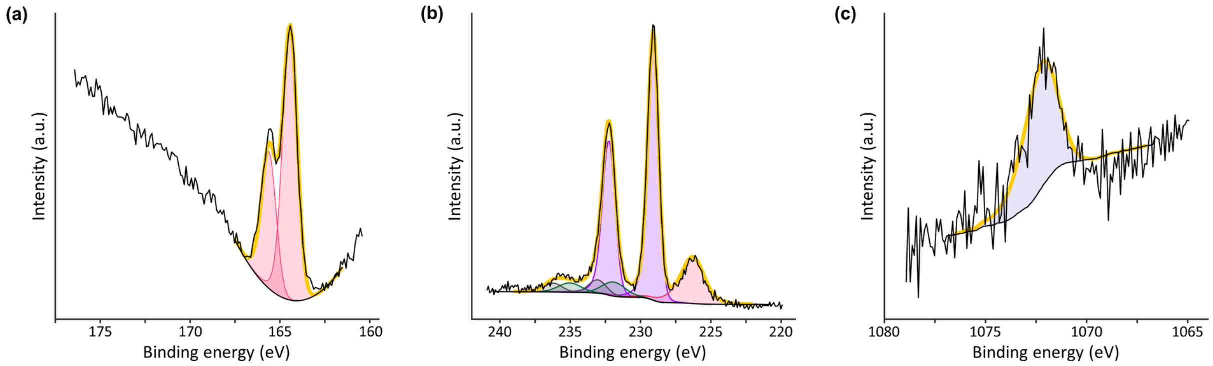

Fig. S21 High-resolution XP-spectra of (a) S 2p, (b) Mo 3d, and (c) Na 1s core level peak regions of the sample Tt9 (750C30M).

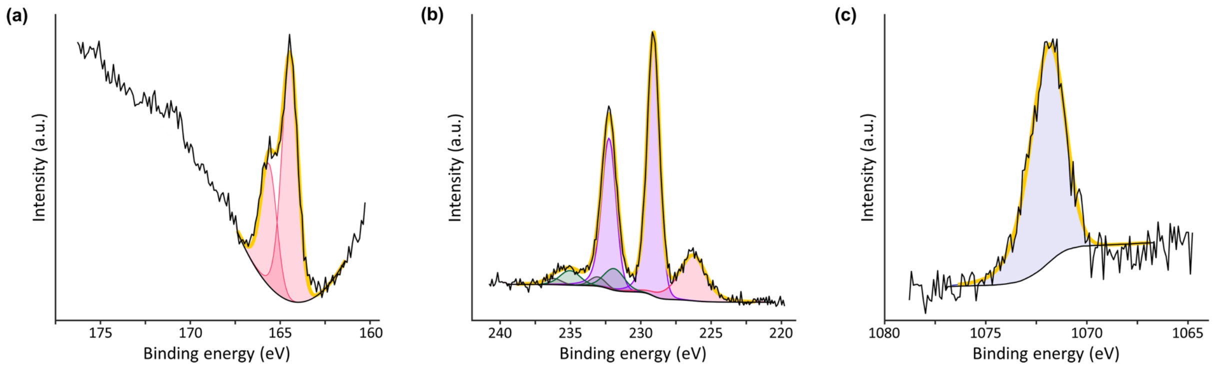

Fig. S20 High-resolution XP-spectra of (a) S 2p, (b) Mo 3d, and (c) Na 1s core level peak regions of the sample O0 (O0M).

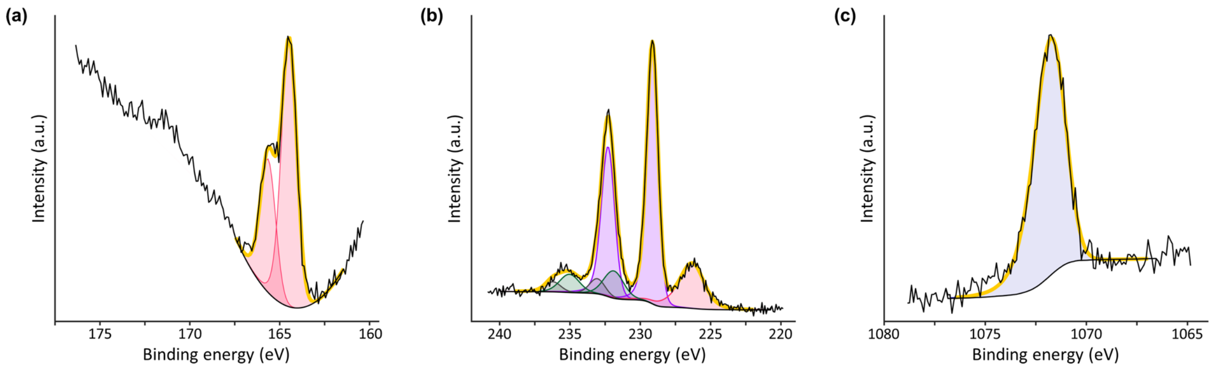

Fig. S22 High-resolution XP-spectra of (a) S 2p, (b) Mo 3d, and (c) Na 1s core level peak regions of the sample O2 (O9M).

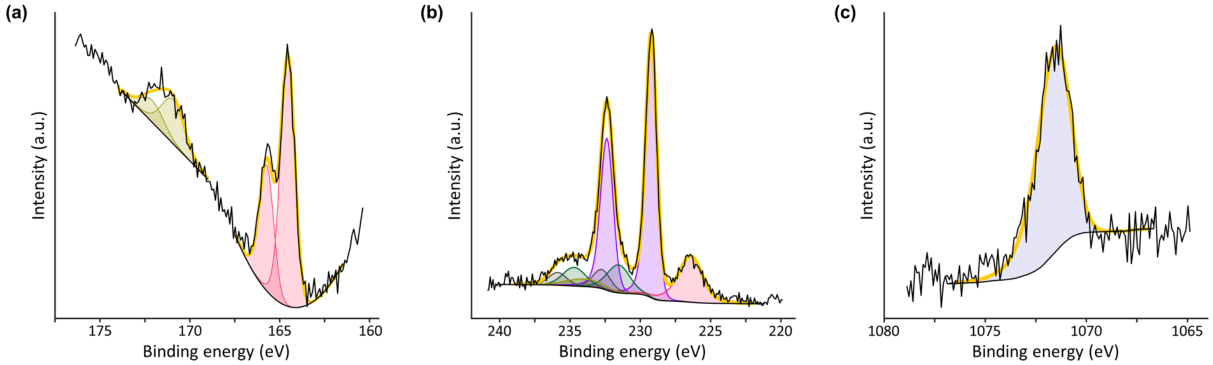

Fig. S23 High-resolution XP-spectra of (a) S 2p, (b) Mo 3d, and (c) Na 1s core level peak regions of the sample O3 (O10M).

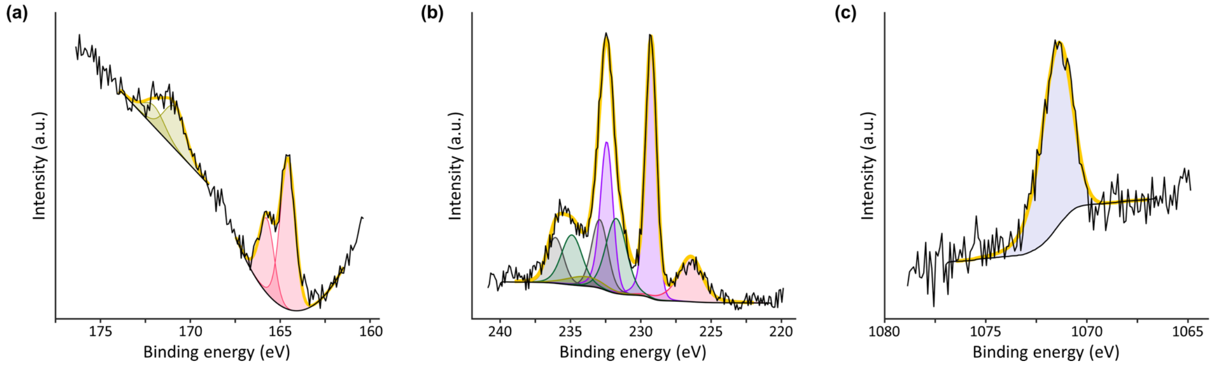

Fig. S24 High-resolution XP-spectra of (a) S 2p, (b) Mo 3d, and (c) Na 1s core level peak regions of the sample O4 (O11M).

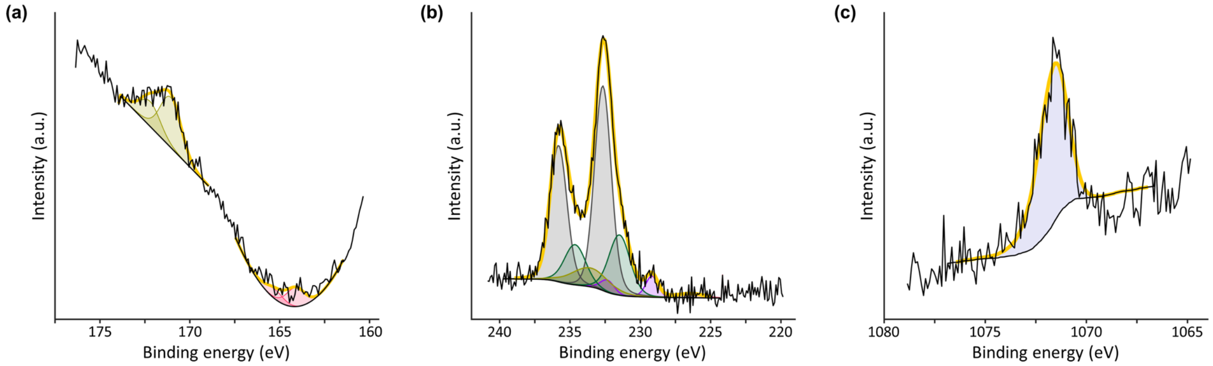

Fig. S25 High-resolution XP-spectra of (a) S 2p, (b) Mo 3d, and (c) Na 1s core level peak regions of the sample O5 (O12M).

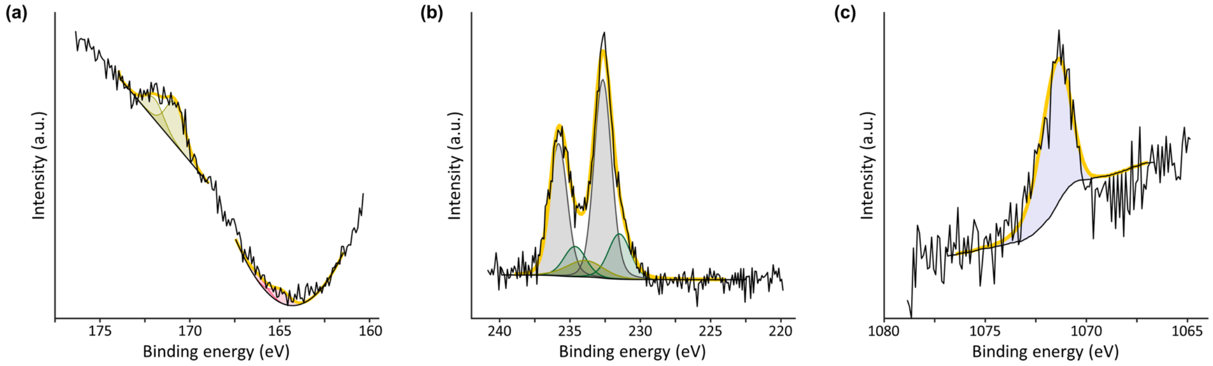

Fig. S26 High-resolution XP-spectra of (a) S 2p, (b) Mo 3d, and (c) Na 1s core level peak regions of the sample O6 (O13M).

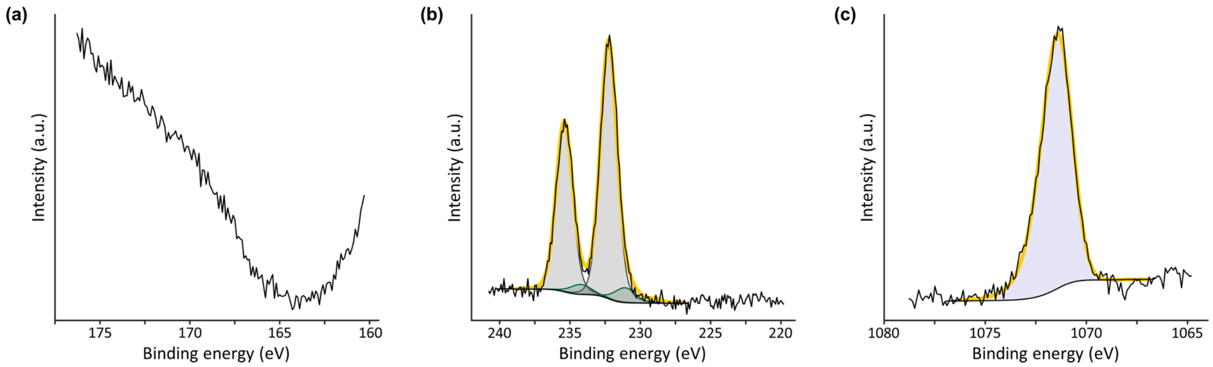

Fig. S28 High-resolution XP-spectra of (a) S 2p, (b) Mo 3d, and (c) Na 1s core level peak regions of the sample S0 (S-O0M).

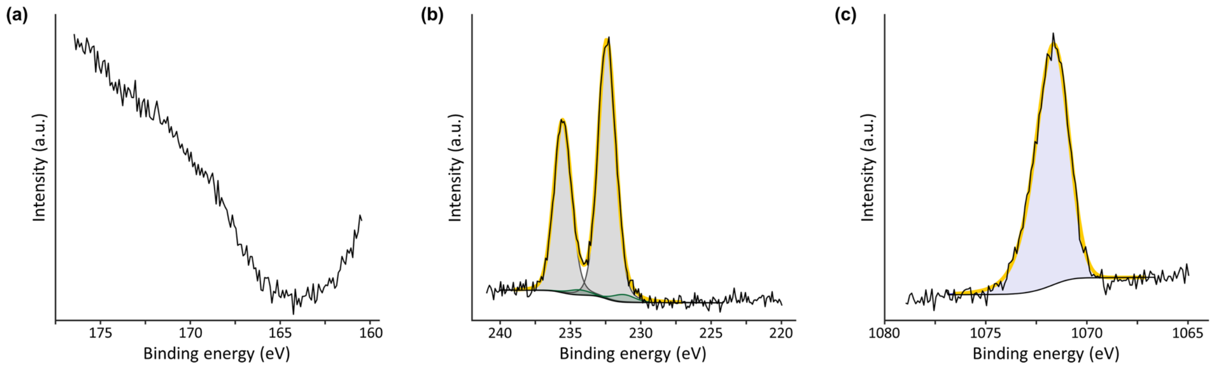

Fig. S27 High-resolution XP-spectra of (a) S 2p, (b) Mo 3d, and (c) Na 1s core level peak regions of the sample S1 (S-O2.5M).

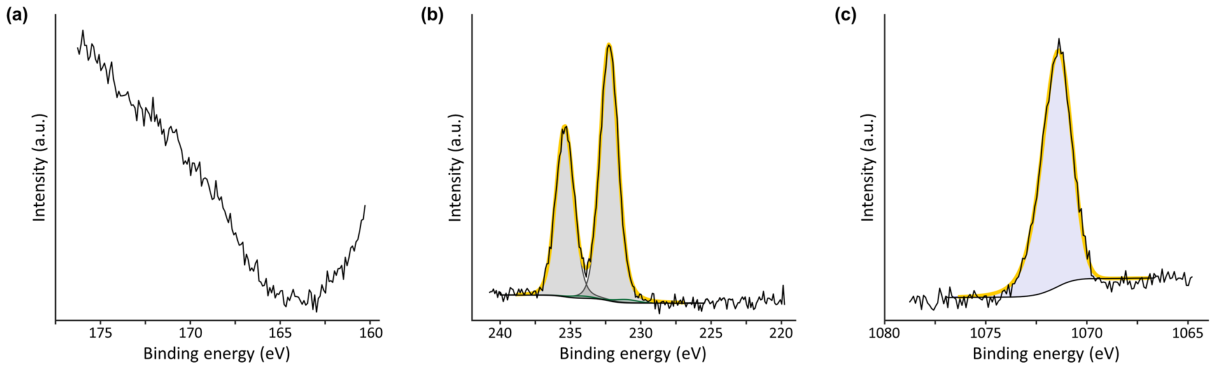

Fig. S29 High-resolution XP-spectra of (a) S 2p, (b) Mo 3d, and (c) Na 1s core level peak regions of the sample S2 (S-O10M).

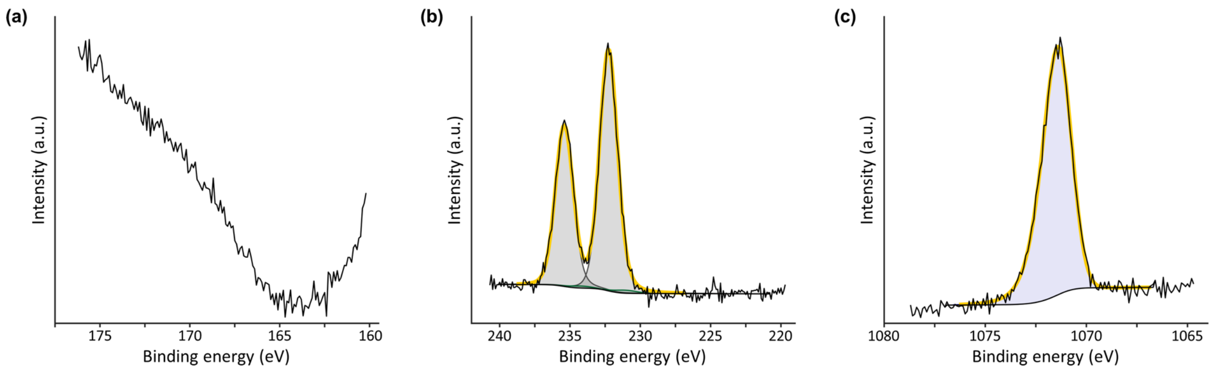

Fig. S30 High-resolution XP-spectra of (a) S 2p, (b) Mo 3d, and (c) Na 1s core level peak regions of the sample S3 (S-O13M).

2.6 Deviation of Apparent Na/Mo-Ratio

The apparent Na/Mo-ratio of the sample SC (spin-coated Na_2MoO_4) is 4.4 and substantially larger than the expected stoichiometry for Na_2MoO_4. As pointed out in *Experimental* in the main manuscript, the quantitative formula used to determine atomic ratios in this work assumes homogeneous composition over the volume probed, which is not the case for our samples. For inhomogeneous compositions, large deviations are expected if the XPS-peaks used for quantification have a substantial difference in binding energy. This applies for the Na/Mo-ratio as the Na 1s and Mo 3d signals differ by about 840 eV in binding energy. An additional contribution to the excess of sodium measured by XPS on spin-coated Na_2MoO_4 might be related to some degree of ionic exchange between the hydrogen and sodium cations at the Si-OH-rich surface of plasma cleaned SiO_2 substrates. By comparison, when the same approach to determine the Na/Mo-ratio is used to estimate the atomic ratio in a homogeneous powder sample, a value of 2.2 is measured, much closer to the expected stoichiometry.

3. Supplementary Figures and Tables

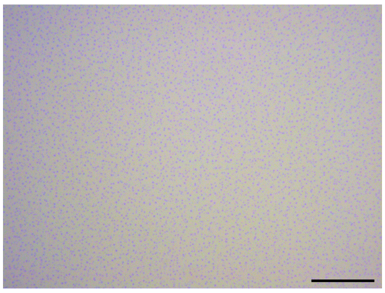

Fig. S31 Light microscopy image of a sample synthesized without O_2 present in the gas flow. The scale bar represents 20 µm.

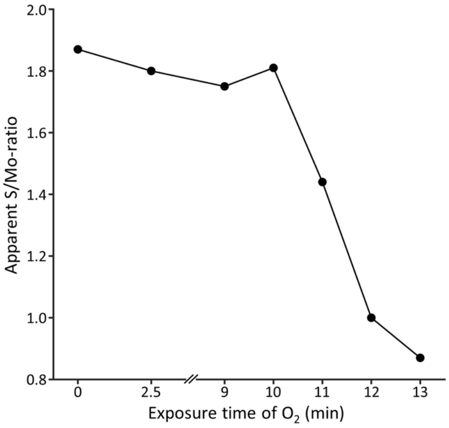

Fig. S32 Apparent S/Mo-ratio, determined by the formula described in the section *Experimental* in the main manuscript of the samples synthesized at 710 °C for 6 min and different exposure times of the O_2.

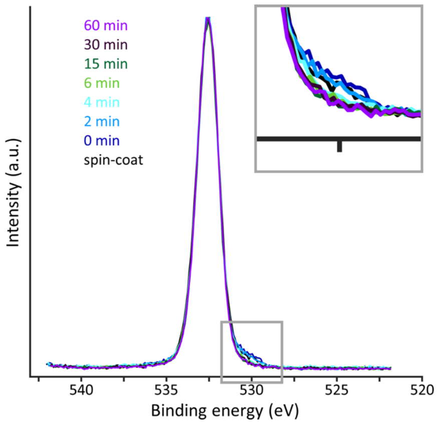

Fig. S33 High-resolution XP-spectra of O 1s core level peak region of samples synthesized at 710 °C and various t_{hold}. The O 1s region is dominated by the signal of the growth substrate used (SiO_2/Si). The inset shows a zoom-in of the shoulder of the O 1s signal to indicate the decrease in the Mo-source (Na_2MoO_4), in line with the appearance of the Mo 3d peaks attributed to MoS_2 (see main manuscript).

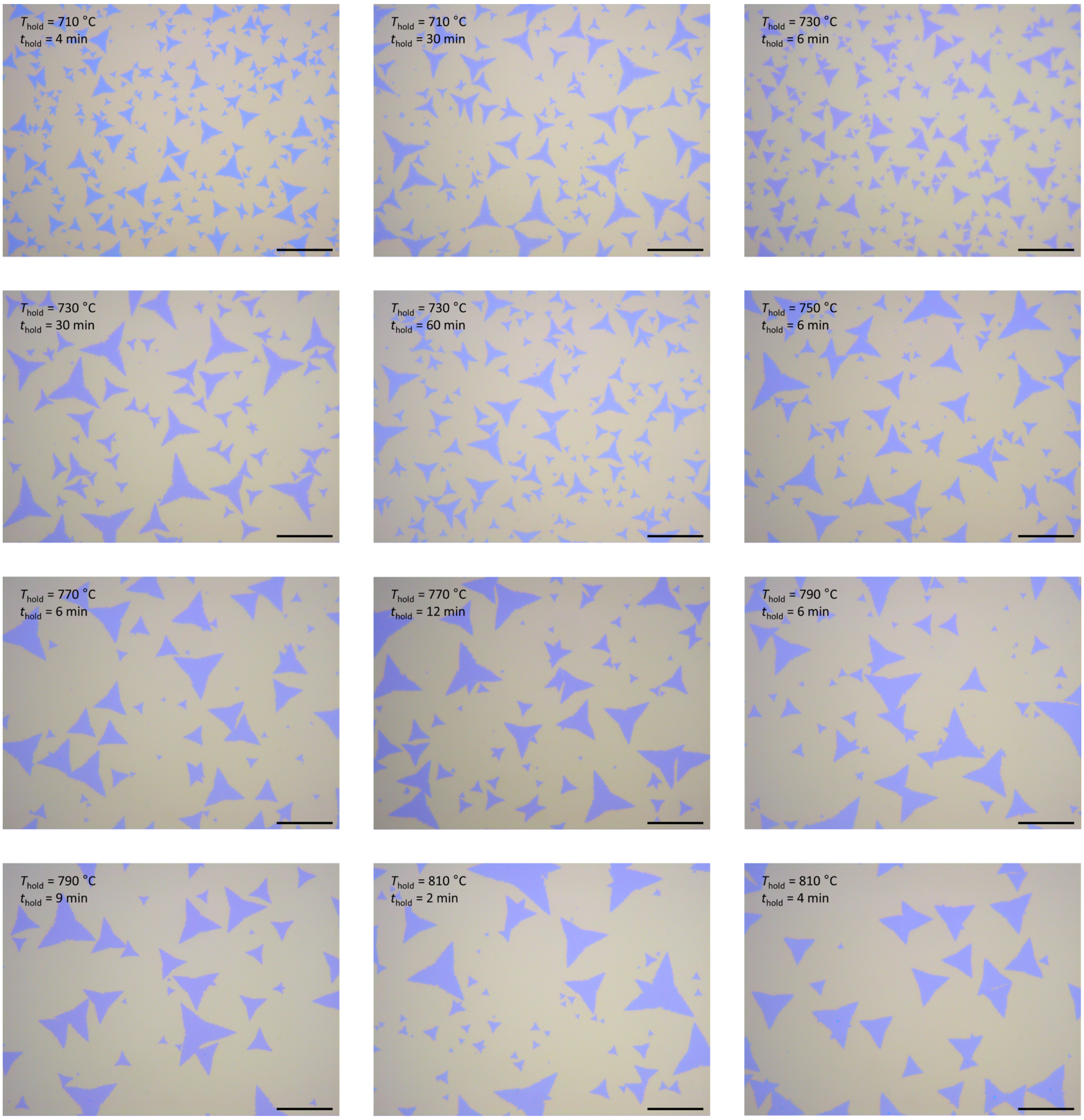

Fig. S34 Light microscopy images of MoS_2 flakes synthesized with different holding temperatures T_{hold} and holding times t_{hold} as indicated directly in the images. Regardless of the *T*-*t*-profiles all synthesized MoS_2 flakes have a triangular shape, show a homogeneous colour, and are evenly distributed over the entire growth substrate. The scale bar represents 20 µm.

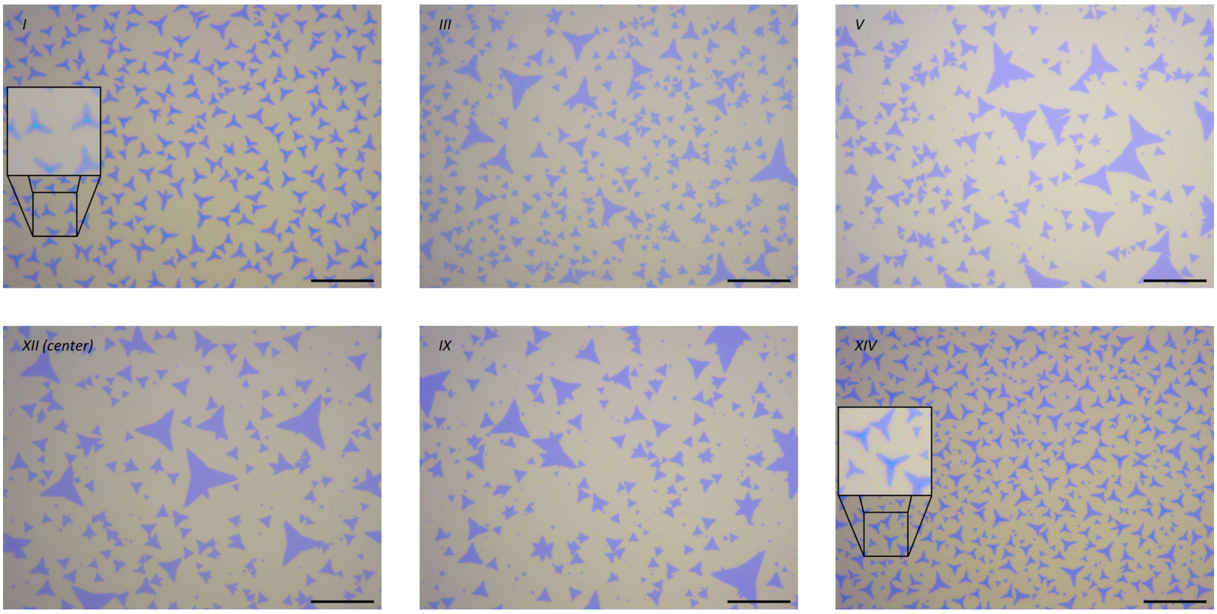

Fig. S35 Light microscopy images of MoS_2 flakes synthesized in the 14-sample test. Regardless of the position of the growth substrate (indicated directly in the images), all synthesized MoS_2 flakes except for the most upstream and most downstream samples (sample *I* and *XIV*) have a triangular shape, show a homogeneous colour, and are evenly distributed over the entire growth substrate. The scale bar represents 20 µm.

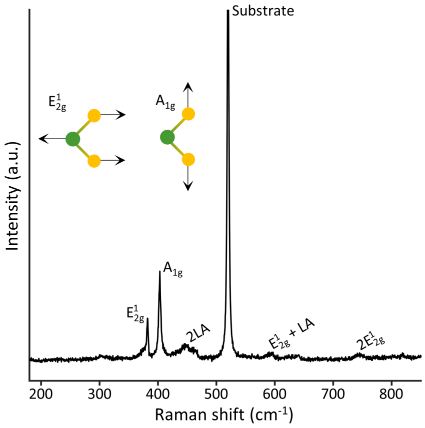

Fig. S36 Raman spectrum of the sample synthesized at 790 °C for 8 min as a representative for a typical Raman spectrum of MoS_2 single layer flakes with the relevant peaks at around 384 and 404 cm^{-1}, representing the $E_{2g}{}^1$ and A_{1g} Raman mode, respectively.

Tab. S5 The difference between the A_{1g} and E_{2g}^1 Raman modes of all synthesized MoS_2 flakes except for the most upstream and most downstream samples (sample *I* and *XIV*) from the 14-sample test is around 20 cm^{-1}, indicating the presence of monolayers.[4,5] For each sample, 4 to 10 different flakes were measured.

**T_{hold} (°C)**	**t_{hold} (min)**	**$A_{1g} – E_{2g}^1$ (cm^{-1})**
710	2	17.7 (±0.4)
	4	17.3 (±0.5)
	6	17.7 (±0.5)
	15	19.1 (±0.4)
	30	20.2 (±0.1)
	60	20.3 (±0.1)
730	2	18.6 (±0.2)
	6	19.1 (±0.2)
	15	20.4 (±0.1)
	30	20.8 (±0.3)
	45	20.6 (±0.1)
	60	20.9 (±0.8)
750	1	18.9 (±0.2)
	3	18.9 (±0.3)
	6	19.0 (±0.5)
	10	19.9 (±0.3)
	15	20.3 (±0.04)
	22	20.9 (±0.5)
	30	20.6 (±0.2)
770	6	20.3 (±0.3)
	10	20.8 (±0.3)
	12	20.7 (±0.5)
	15	20.5 (±0.3)
790	2	19.3 (±0.1)
	6	20.7 (±0.5)
	8	20.9 (±0.4)
	9	20.5 (±0.2)
	10	19.9 (±0.1)
810	2	19.5 (±0.3)
	4	20.9 (±0.2)
	6	20.4 (±0.4)
	7	20.1 (±1.1)

**Number of sample in the 14-sample test**	**$A_{1g} – E_{2g}^1$ (cm^{-1})**
II	20.3 (±0.3)
III	20.2 (±0.6)
IV	20.8 (±0.3)
V	20.2 (±0.4)
VI	20.2 (±0.6)
VII	20.5 (±0.2)
VIII	20.3 (±0.3)
IX	20.3 (±0.3
X	20.8 (±0.4)
XI	20.3 (±0.5)
XII	20.6 (±0.9)
XIII	19.7 (±0.2)

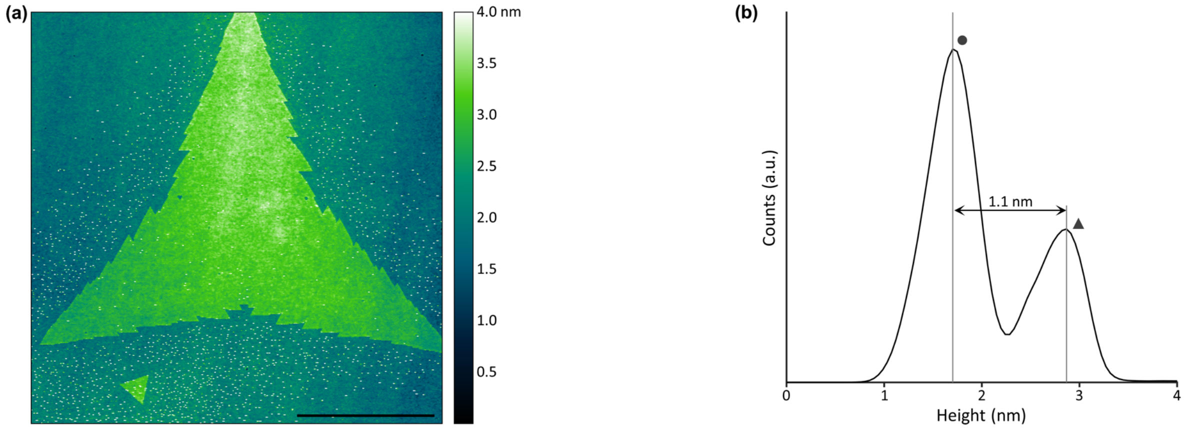

Fig. S37 (a) AFM height image and (b) statistical height analysis of MoS_2 flakes synthesized at 750 °C for 15 min as a representative for the height measurements based on the description in [6]. The left peak (●) represents the substrate and the right peak (▲) the MoS_2 flakes. The scale bar represents 5 µm.

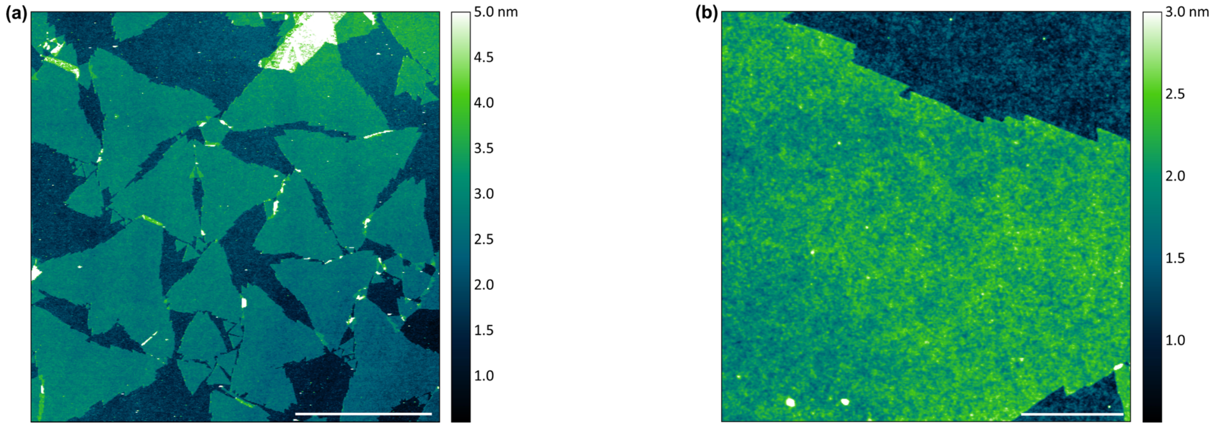

Fig. S38 AFM images of MoS_2 flakes synthesized at 710 °C for 6 min and picked-up by a SiO_2/Si substrate while floating on the water surface after being detached (transfer by the pick-up method, see *Experimental* in the main manuscript). The scale bar represents (a) 5 µm and (b) 500 nm.

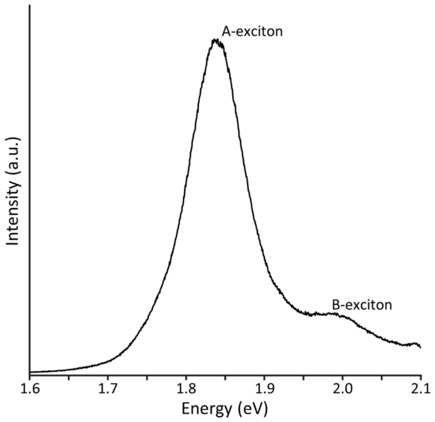

Fig. S39 Photoluminescence (PL) spectrum of the sample synthesized at 790 °C for 6 min as a representative for the PL spectrum of a MoS_2 single layer flake with its characteristic peaks at around 1.85 and 2.0 eV, representing the A- and B-exciton, respectively.

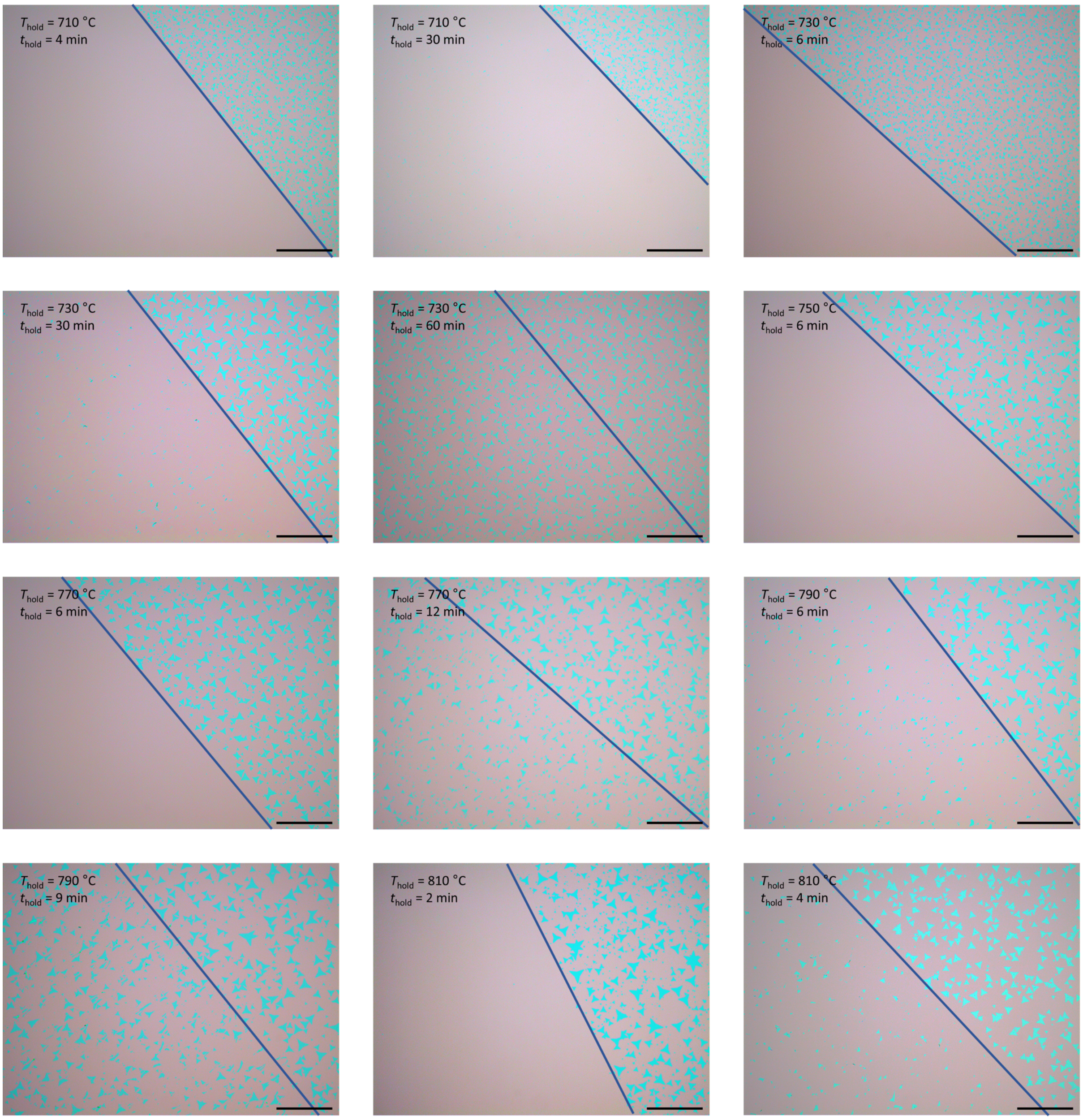

Fig. S40 Light microscopy images of MoS_2 flakes synthesized with different holding temperatures T_{hold} and holding times t_{hold} as indicated directly in the images to show the influence of the *T*-*t*-profiles on the detachability of the synthesised MoS_2 flakes. The blue lines mark the immersion depth of the growth substrates into water to detach the MoS_2 flakes. The scale bar represents 100 µm.

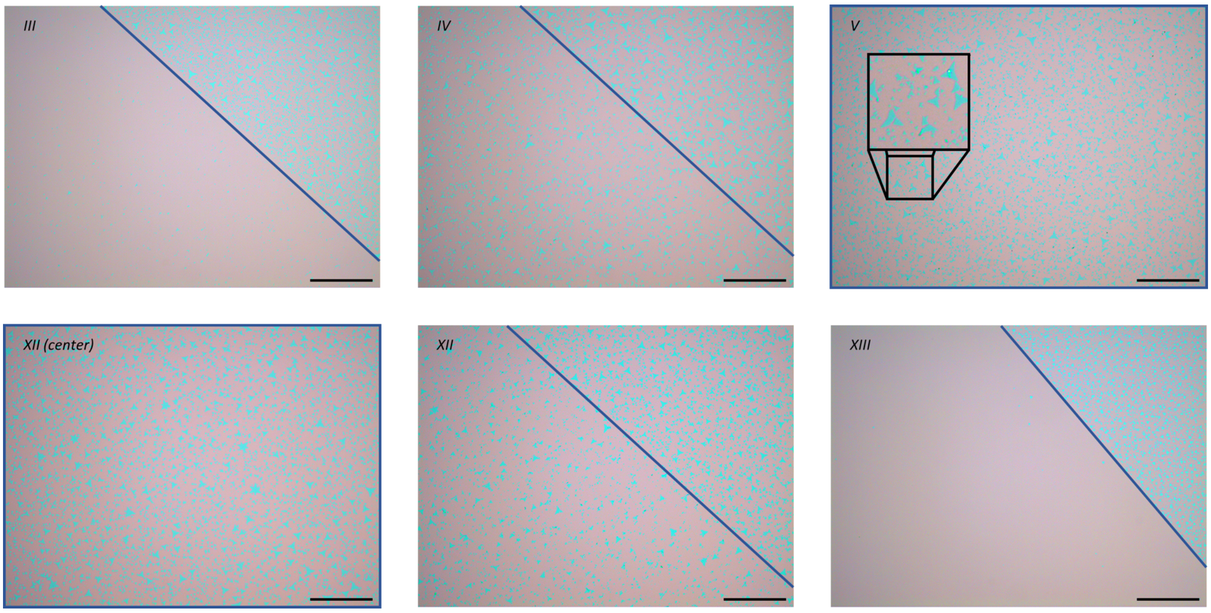

Fig. S41 Light microscopy images of MoS_2 flakes synthesized in the 14-sample test to show the influence of the position of the growth substrate in the tube furnace and, therefore, how the *T*-*t*-profile influences the detachability of the synthesised MoS_2 flakes. The blue lines mark the immersion depth of the growth substrates in water to detach the MoS_2 flakes. Samples *V* and *VII* were completely immersed in water. The scale bar represents 100 µm.

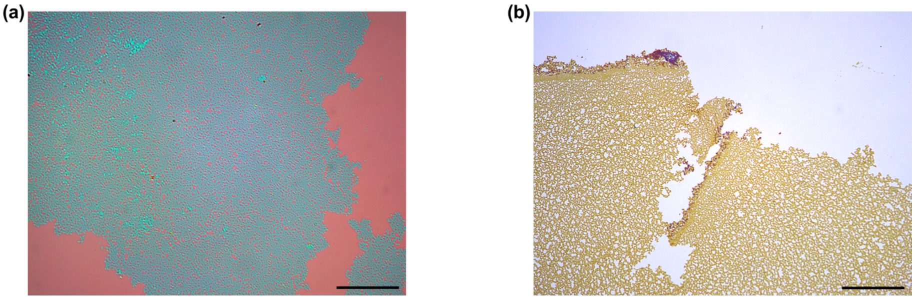

Fig. S42 MoS_2 flakes picked-up after detaching with a (a) SiO_2/Si and (b) gold substrate. The scale bar represents 200 µm.

(a)

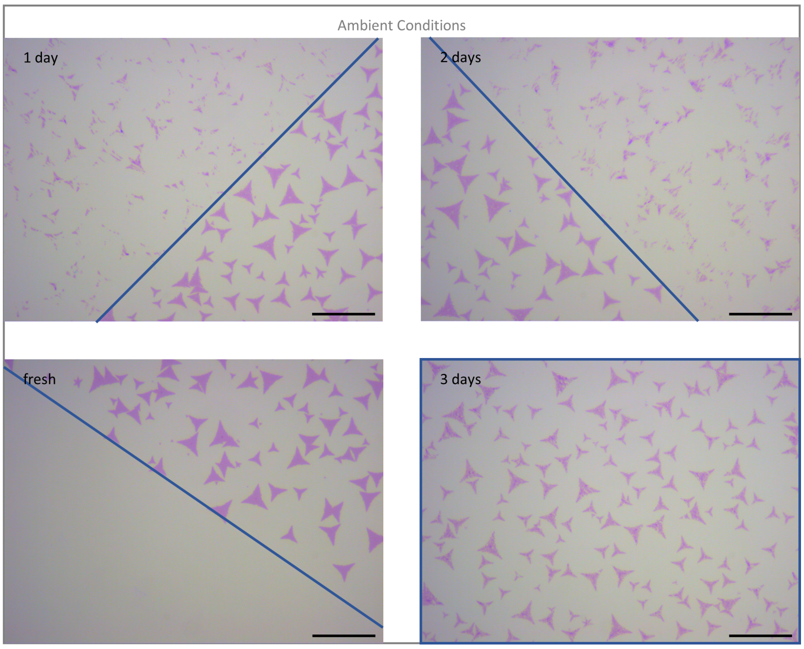

(b)

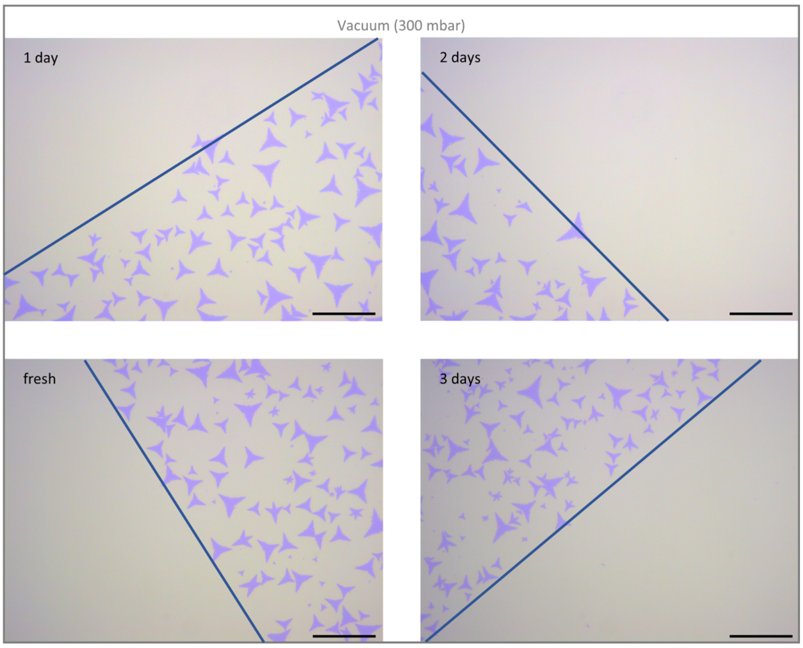

Fig. S43 Light microscopy images of MoS_2 flakes stored (a) at ambient conditions and (b) in vacuum (300 mbar) for different storage times as indicated directly in the images to test the long-term stability of the MoS_2 flakes detachability. The blue lines mark the immersion depth of the growth substrate in water. Samples without a blue line were immersed completely in water. The scale bar represents 20 µm.

(c)

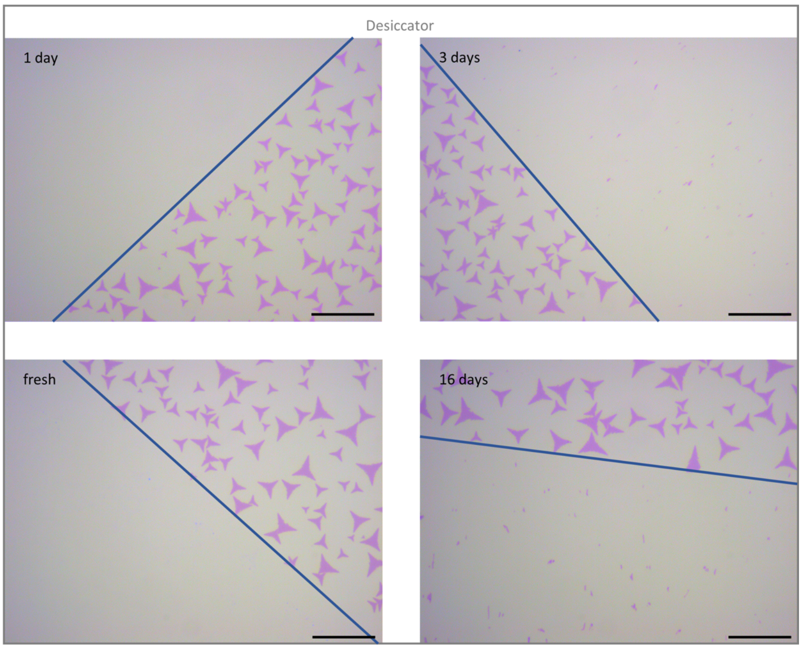

Fig. S43-Continuation Light microscopy images of MoS_2 flakes stored in (c) a desiccator for different storage times as indicated directly in the images to test the long-term stability of the MoS_2 flakes detachability. The blue lines mark the immersion depth of the growth substrate in water. The scale bar represents 20 µm.

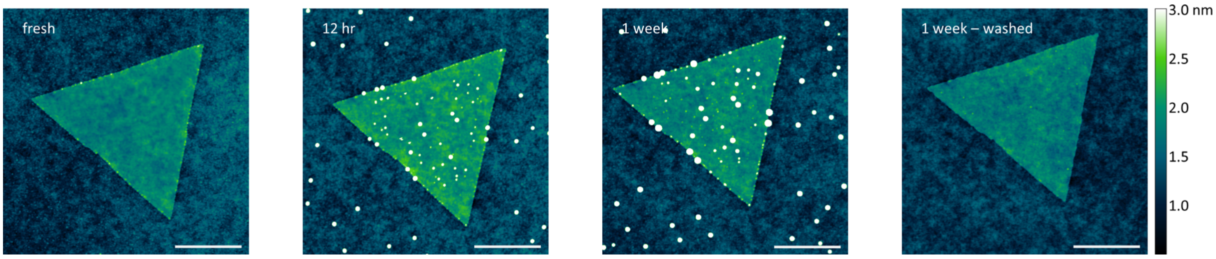

Fig. S44 AFM measurements of a sample synthesized at 770 °C for 12 min and after different storage time. The scale bar represents 500 nm.

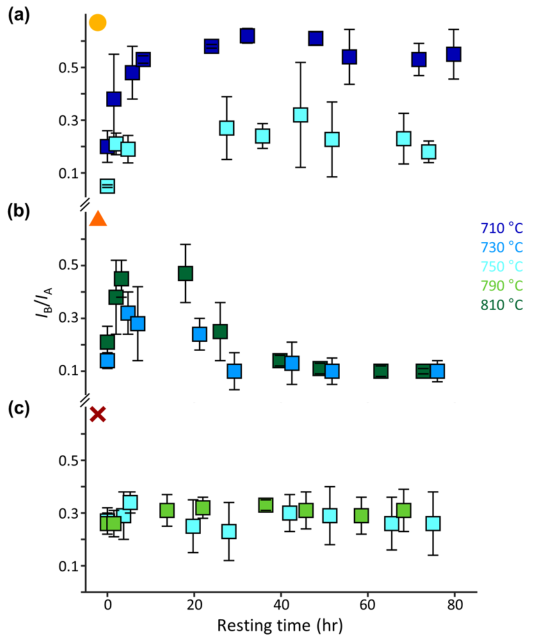

Fig. S45 I_B/I_A-ratio versus resting time of samples synthesized with different *T*-*t*-profiles, where different colors indicate the corresponding T_{hold}. The samples in (a) are 100 % detachable (yellow circle), (b) are partial detachable (orange triangle), and (c) are not detachable at all (red cross).

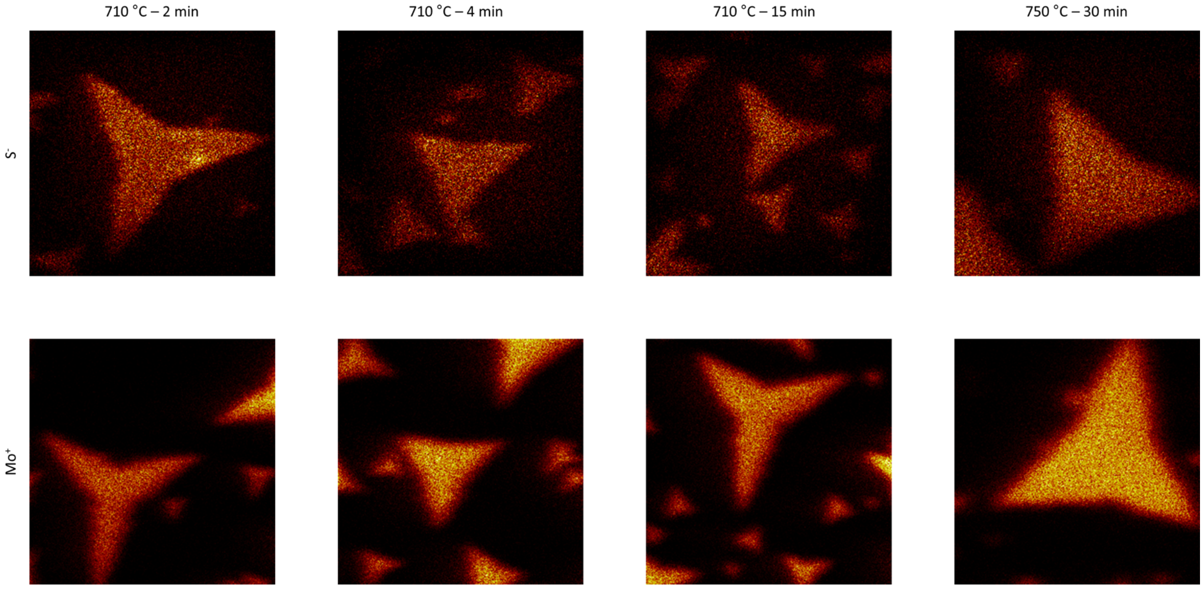

Fig. S46 Elemental mapping of S^--and Mo^+-ions of samples synthesized with different *T*-*t*-profiles measured with ToF-SIMS operated in negative (top row) and positive (bottom row) mode.

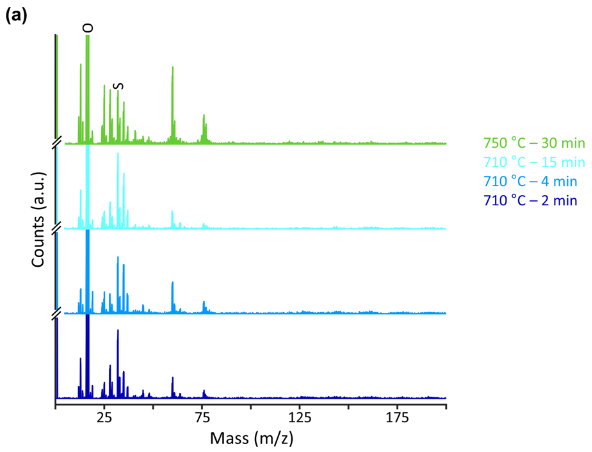

Fig. S47 ToF-SIMS spectra of samples synthesized with different *T*-*t*-profiles, with ToF-SIMS operated in (a) negative and (b) positive mode.

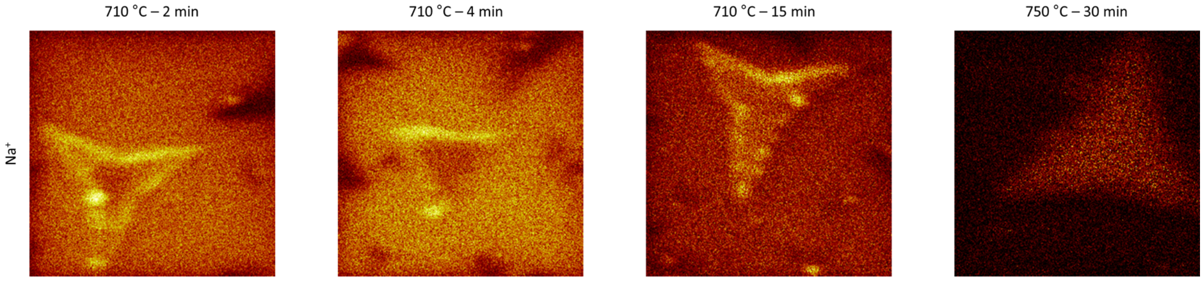

Fig. S48 Elemental mapping of Na^+-ion of samples synthesized with different *T*-*t*-profiles measured with ToF-SIMS operated in positive mode.

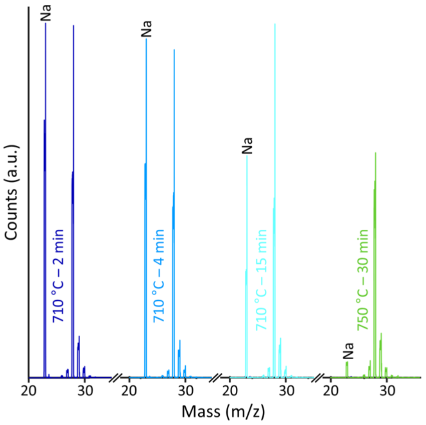

Fig. S49 ToF-SIMS spectra operated in the positive mode indicating decreased counts for Na^+ for samples with increasing T_{hold} and t_{hold}.

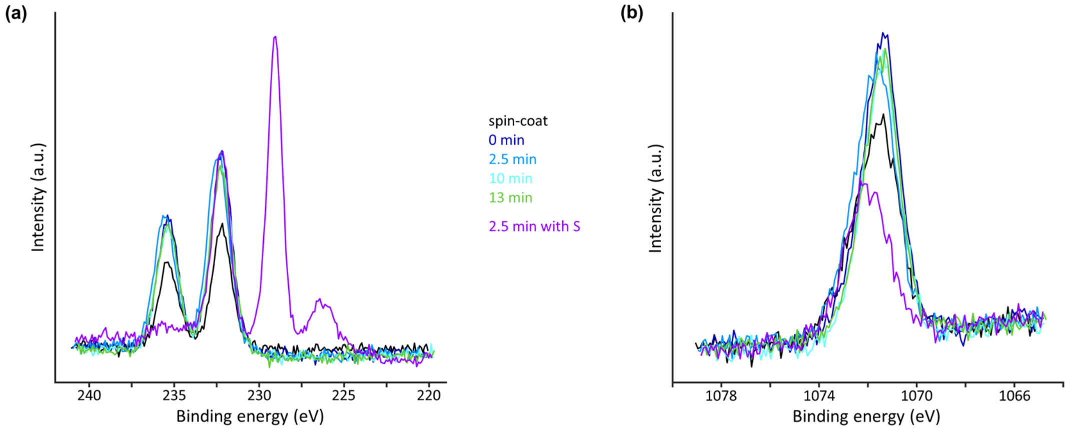

Fig. S50 High-resolution XP-spectra of (a) Mo 3d and (b) Na 1s core level peak regions of samples treated at 710 °C for 6 min in absence of sulfur and different exposure times of O_2. As a reference, the sample synthesized at 710 °C for 6 min with sulfur present is shown in purple.

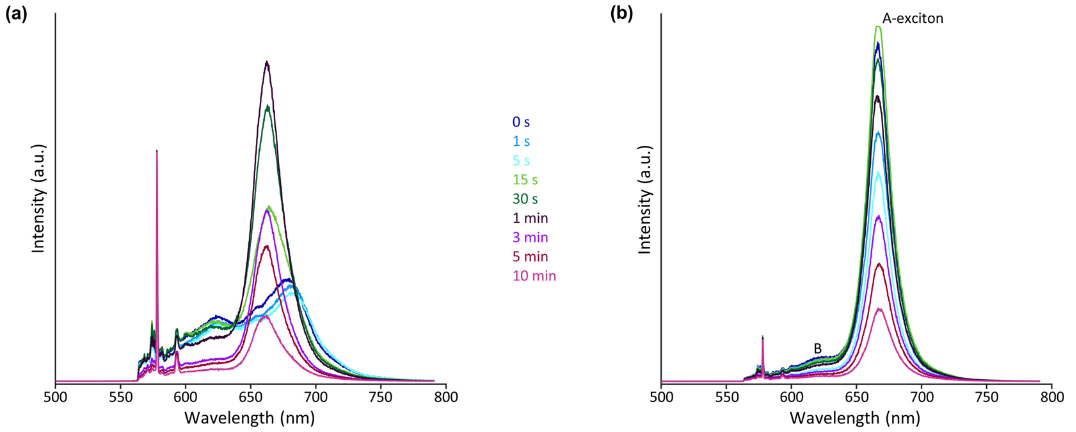

Fig. S51 (a) Photoluminescence (PL) spectra of a MoS_2 flake after transfer onto a fresh SiO_2/Si substrate to eliminate any influence of the growth substrate on the PL measurement. The spectrum changes depending on the laser irradiation time (561 nm, 7.6 mW) prior to PL measurement due to removal of adsorbed water and PMMA residues of the transfer.[8] (b) PL spectra of a MoS_2 flake on the growth substrate. The spectra only change in intensity with increasing laser irradiation, while the intensity ratio of the B-/A-exciton remains constant at 0.06, corroborating that laser irradiation for cleaning of the transferred flake has no detrimental effects.

4. References

1 J. Kong, K.T. Park, A.C. Miller and K. Klier, *Surface Science Spectra*, 2000, **7**, 69
2 J.D.P. Counsell, S.J. Coultas and N. Gerrard, *Surface Science Spectra*, 2021, **28**, 024007
3 D.S. Jensen, S.S. Kanyal, N. Madaan, M.A. Vail, A.E. Dadson, M.H. Engelhard, M.R. Linford, *Surface Science Spectral*, 2013, **20**, 36
4 H. Li, Q. Zhang, C.C.R. Yap, B.K. Tay, T.H.T. Edwin, A. Olivier and D. Baillargeat, *Adv. Funct. Mater.*, 2012, **22**, 1385
5 E. Pollmann, L. Madauss, S. Schumacher, U. Kumar, F. Heuvel, C. Vom Ende, S. Yilmaz, S. Güngörmüs and M. Schleberger, *Nanotechnology*, 2020, **31**, 505604
6 Y. Xiao, W. Zhend, B. Yuan, C. Wen and M. Lanza, *Cryst. Res. Technol.*, 2021, **56**, 2100056
7 S.S. Chou, Y.-K. Huang, J. Kim, B. Kaehr, B.M. Foley, P. Lu, C. Dykstra, P.E. Hopkins, C. Brinker, J. Huang and V.P. Dravid, *J. Am. Chem. Soc.*, 2015, **137**, 1742
8 C. Hou, J. Deng, J. Guan, Q. Yang, Z. Yu, Y. Lu, Z. Xu, Z. Yao and J. Zheng, *Phys. Chem. Chem. Phys.*, 2021, **23**, 24579
